# Targeted Isolation of Indole Alkaloids from *Streptomyces* sp. CT37

**DOI:** 10.3390/molecules25051108

**Published:** 2020-03-02

**Authors:** Qing Fang, Fleurdeliz Maglangit, Morgane Mugat, Caroline Urwald, Kwaku Kyeremeh, Hai Deng

**Affiliations:** 1 Marine Biodiscovery Centre, Department of Chemistry, University of Aberdeen, Meston Walk, Aberdeen AB24 3UE, Scotland, UK; r01qf16@abdn.ac.uk (Q.F.); r01fm16@abdn.ac.uk (F.M.); 2 Department of Biology and Environmental Science, College of Science, University of the Philippines Cebu, Lahug, Cebu City 6000, Philippines; 3 ENSAIA, 2 avenue de la forêt de Haye, 54505 vandœuvre lès Nancy, France; morgane.mugat@orange.fr (M.M.); carolinurwald@gmail.com (C.U.); 4 Department of Chemistry, University of Ghana, P.O. Box LG56 Legon-Accra, Ghana; kkyeremeh@ug.edu.gh

**Keywords:** legonimide, indole alkaloids, imide, *Streptomyces* sp. CT37, natural products, antifungal

## Abstract

Four compounds (**1**–**4**) were isolated from the extracts of *Streptomyces* sp. CT37 using bioassay in conjunction with mass spectrometric molecular networking (MN) driven isolation. Their complete structures were established by high-resolution electrospray ionization mass spectrometry (HR-ESIMS), and 1D and 2D nuclear magnetic resonance (NMR) data. Legonimide **1** was identified as a new alkaloid containing a rare linear imide motif in its structure, while compounds **2–4** were already known and their structures were elucidated as 1H-indole-3-carbaldehyde, actinopolymorphol B, (*2R,3R*)-1-phenylbutane-2,3-diol, respectively. The biosynthetic pathways of **1–4** were proposed based on the reported biogenesis of indole alkaloids in literature. Bioactivity tests for **1** and **2** revealed moderate growth inhibition activity against *Candida albicans* ATCC 10231 with MIC_95_ values of 21.54 µg/mL and 11.47 µg/mL, respectively.

## 1. Introduction

Microbial natural products (NPs) encompass several chemical structures that constitute a treasure trove of high-value molecules, such as antibiotics, anticancer and antioxidant drugs [1]. *Streptomyces* a prominent resource for natural product discovery contributing up to 75% of present day clinically used antibiotics [2,3]. As part of the efforts of our laboratory to investigate microbial NPs with antibiotic bioactivity from under-investigated environments, we have studied several *Streptomyces* isolates from soil samples collected in Legon, Ghana [4,5,6,7,8,9,10,11,12,13,14].

Recently, metabolomics guided microbial NPs discovery processes have been widely used for the prediction of bioactive metabolite structures and prioritization of extracts for isolations [15,16]. The method offers a route to deliver qualitative and quantitative data analysis of chemical space for the metabolites present in complex bacteria fermentation extracts [9]. A useful analytical technique that has been integrated into the metabolomics-guided microbial NPs discovery is HR-ESIMS and associated MS/MS fragmentation data. However, the complexity of HR-ESIMS/MS data prevents manual annotation of the presence of metabolites in the extracts. As such, several computing-based data mining tools have been applied; one of the widely used is the Molecular Networking (MN) database [17,18,19,20]. The MN database is a web-based server that is extremely useful for dereplicating natural product structures by comparing both experimental HR-ESIMS (MS/MS) spectra with the database [17]. The method is useful for the effective dereplication and annotating of specialised metabolite (SM) families and individual molecules. 

The current study establishes mass spectrometric molecular networking (MN) in conjunction with bioassay tests to guide isolation of the bioactive components from the Ghanaian isolate, *Streptomyces* sp. CT37. The targeted isolation afforded four compounds: legonimide **1**, 1H-indole-3-carbaldehyde **2**, actinopolymorphol B **3**, and (2*R*,3*R*)-1-phenylbutane-2,3-diol **4**.

## 2. Results and Discussion

Chemical profiling of the *Streptomyces* sp. CT37 strain was carried out in different cultivation media [21]. Eight different fermentation broths (ISP2-ISP7, modified Bennett’s, starch casein, Table S1) were selected based on the recommended medium for *Streptomyces* species, which differ with respect to carbon source and salt concentration [22]. The extracts obtained in the eight broths were subjected to disc diffusion bioassay tests using a panel of Gram-positive, Gram-negative, and fungal pathogens (Table S2). Only the ISP2 extract showed inhibition zone (9.0 mm) against *Candida albicans* ATCC 10,231 (Figure S1). Subsequently, large-scale fermentation (6.0 L) was carried out in ISP2 medium, followed by solid phase extraction (SPE) to yield six fractions, S1–S6. HRESIMS and molecular network analyses of the fractions identified five major compound families: indole alkaloids, phenols, surfactins, deferoxamines, phthalates, and cyclopeptides (Figure S2). Manual dereplication [23] using MS^1^ spectra of compounds against available databases (AntiBase, The Natural Products Atlas [24]) identified fraction S2 to harbor alkaloid metabolites that were not previously reported in literature (Table S3). Disc diffusion assay of S2 against *C. albicans* ATCC 10,231 revealed zone of inhibition that was not observed in other fractions. Hence, S2 was further purified by high pressure liquid chromatography (HPLC) and led to the isolation of four compounds, one of which is a new alkaloid bearing a linear imide motif, legonimide **1** (2.1 mg), and three known compounds: **2** (1.1 mg), **3** (1.0 mg), and **4** (0.8 mg). 

### 2.1. Structure Elucidation 

**Figure 1:**
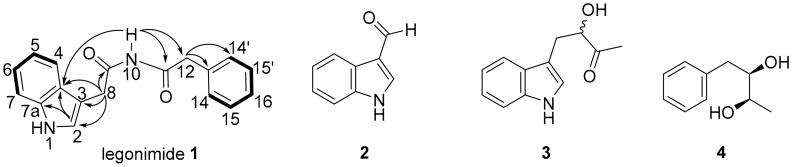
Compounds **1–4** isolated from *Streptomyces* sp. CT37 ISP2 extract; compound **1** with correlation spectroscopy (COSY) (**―**) and key heteronuclear multiple bond correlation (HMBC) (→) correlations.

The structures of compounds **1**–**4** were established by analysis of the HRESIMS, 1D, and 2D NMR data and by comparison with the reported data in literature [25,26,27] (Figure 1). 

Compound **1** was isolated as a pale yellow amorphous solid. The HR-ESIMS spectrum of **1** exhibited a prominent peak at *m/z* 293.1280 [M + H]^+^ corresponding to the molecular formula of C_18_H_16_N_2_O_2_ (*calcd* for C_18_H_17_N_2_O_2_^+^ 293.1285, ∆ = −1.70 ppm) possessing 12 degrees of unsaturation.

The ^1^H nuclear magnetic resonance (NMR) spectrum in DMSO-*d6* (Table 1) of **1** indicated the presence of two exchangeable NH protons (δ_H_ 7.37, 10.96), two sp^3^ methylenes (δ_H_ 3.35, 3.46), nine aromatic methines (δ_H_ 7.54, 7.34, 7.31, 7.28, 7.28, 7.21, 7.21, 7.06, and 7.02), and one sp^2^ methine (δ_H_ 7.18). In addition to the signals corresponding to the above carbons, analysis of the ^13^C NMR and heteronuclear single quantum coherence (HSQC) spectra revealed the presence of six quaternary carbons, including two carbonyls (δ_C_ 173.1, 172.3) and four aromatic groups (δ_C_ 136.6, 136.5, 127.3, and 109.2).

Thorough inspection of the 2D NMR spectra disclosed the presence of 4 substructures (A-D) (Figure S3), including a 3H-subsituted indole, acetyl group (B), acetamide subunit (C), and one monosubstituted aromatic ring (D) in the structure of **1**. The monosubstituted phenyl group and the ring in the indole moiety comprised two separate spin systems, H-14 through H-14′ and H-4 through H-7, respectively, as indicated in the ^1^H-^1^H correlation spectroscopy (COSY) spectrum. The heteronuclear multiple bond correlation (HMBC) from H-8 (δ_H_ 3.46) to the carbonyl carbon at C-9 (δ_C_ 173.1), and NH-10 (δ_H_ 7.37) to carbonyl C-11 (δ_C_ 172.3) and methylene carbon at C-12 (δ_C_ 42.0), established the presence of the acetyl and acetamide subunits, respectively. The HMBC correlation from H-12 (δ_H_ 3.35) to C-13 (δ_C_ 136.6) and C-14 (δ_C_ 128.1) established the connectivity of substructure C to D at C-13. The cross peaks from H-8 to C-2 (δ_C_ 123.6), C-3 (δ_C_ 109.2), and C-4a (δ_C_ 127.3) confirmed the connection of the indole subunit (substructure A) to the acetyl group (substructure B) at C-3. The structure of **1** was further corroborated by the long-range HMBC correlation from NH-10 to C-4a and C-12; thereby, the imide group flanked the 3H-substituted indole moiety and the monosubstituted benzene ring (Figures S9–S11). Hence, the structure of **1** was elucidated representing a new alkaloid containing a linear imide motif as *N*-(2-(1H-indol-3-yl)acetyl)-2-phenylacetamide and named as legonimide in association with Legon, Ghana. 

The free imide group in **1** can adopt three conformations: *cis-trans*, *trans-trans* and *cis-cis* [28] (Figure S12). There was no HMBC correlation from NH-10 to C-8, while correlation was observed from NH-10 to C-12 (Figure S13), suggesting that the imide motif in **1** is likely a *cis-trans* conformer. This observation is in agreement with those reported for imide-containing natural products that the *cis-trans* conformation is the most stable [29,30,31,32].

Compound **2** was identified as 1H-indole-3-carbaldehyde previously isolated from plants [33], marine sponge [34]*,* and several *Streptomyces* species [35,36] (Figures S14–S18, Table S4). Compound **3** was consistent with the reported data for actinopolymorphol B from *actinopolymorpha rutilus* [27] (Figures S19–S23, Table S4). Compound **4** was 1-phenylbutane-2,3-diol found in several plants [37]*. A threo* isomer of **4** was previously isolated from a *actinomycete Williamsia* sp. MCCC 1A11233 (Figures S24–S28) [38]. The configuration of **4** was assigned upon careful comparison of the NMR data with those reported for the four synthetic stereoisomers, two *threo* (2*S*,3*R* and 2*R*,3*S*) and two *erythro* (*2S,2S* and *2R,3R*) [26,37] (Table S5). The NMR data of **4** was consistent with the reported data for *threo* isomers. Furthermore, comparison of the optical rotation of **4** with the synthetic stereoisomers pointed to (*2R,3R*)-1-phenylbutane-2,3-diol [26,37] (Tables S5 and S6). However, we could not exclude the possibility that compound **4** could be a mixture of enantiomers.

### 2.2. Proposed Biosynthesis Pathway of **1**–**4**

**Figure 2:**
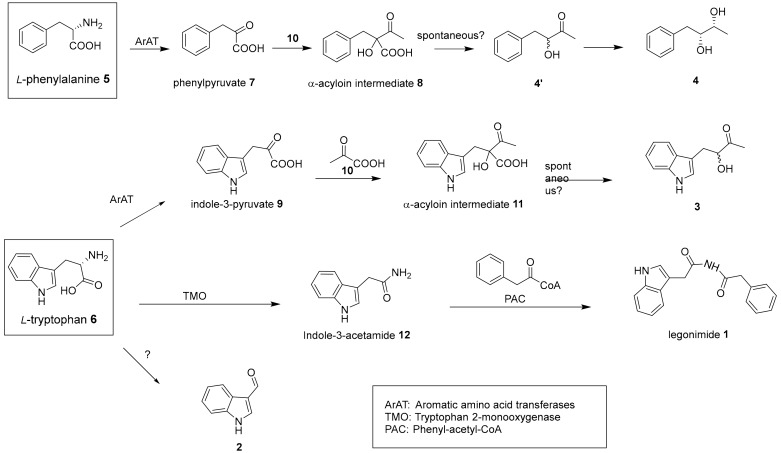
Proposed biosynthesis pathway of **1**–**4.**

Based on the reported biogenesis of indole alkaloids from bacteria [11,13,35,36,37,38], we proposed the biosynthesis pathways of compounds **1–4** (Figure 2). Inspection of the structure of legonimide **1** led us to speculate that it is biosynthesized from the condensation reaction between the phenyl-acetyl-CoA, a common intermediate, and the indole-3-acetamide **12**, an oxidative product of l-tryptophan **6** catalysed by tryptophan 2-monooxygenase [39]. Indole-3-carbaldehyde **2** was found as part of pathogen defense in cruciferous plants. Biochemical studies indicated that **2** is synthesized from tryptophan **6** via the intermediate indole-3-acetonitrile by a cytochrome P450 enzyme CYP71B6 in *Arabidopsis thaliana* [40]. Compound **2** is also a metabolite produced by human gastrointestinal bacteria, particularly species of the *Lactobacillus* genus [41] and various *Streptomyces* species [35,36]. However, the enzyme(s) responsible for the production of **2** from bacterial origins remain poorly understood. Compounds **3** and **4** may derive from the carboligation reaction between pyruvate and indole-3-pyruvate **9** or phenylpyruvate **7** catalysed by a thiamin-diphosphate dependent enzyme to yield α-hydroxyl acyloins **8** and **11**, respectively [11,13]. Possible spontaneous decarboxylation of **8** and **11** could lead to the production of racemers **3** and the intermediates **4′**. One of the racemic **4′** may be further reduced into **4** [11]. Interestingly, analysis of GNPS network allowed the identification of ions likely correlated to four intermediates (7, 4′, 9, and 12) in indole alkaloids and phenyls clusters, suggesting the proposed biosynthetic pathways may be plausible. However, the rest of the proposed intermediates cannot be observed, possibly due to their instable natures with the tendency of degradation. 

### 2.3. Biological Test 

The activity of **1**–**4** was evaluated against Candida albicans ATCC 10,231 (Table 2). Compounds **1** and **2** showed moderate activity with minimum inhibitory concentration (MIC) values of 21.54 μg/mL and 11.47 μg/mL, respectively (Figure S29), while no activity was observed for **3** and **4** at the highest concentration tested (50 μg/mL). 

## 3. Experimental

### 3.1. General Experimental Procedures

HR-ESIMS data were obtained in positive ESI mode with a mass range of 100–2000 *m/z* (maximum resolution 30,000) on a Thermo Scientific MS system (LTQ XL/LTQ Orbitrap Discovery, Waldbronn, Germany). Reserpine (*m/z* 609.2807) was used as a lock mass for internal calibrant during data acquisition. The following instrument parameters were used: capillary voltage 45 V, spray voltage 4.5 kV, capillary temperature 200 °C, auxiliary gas flow rate 10–20 arbitrary units, and sheath gas flow rate 5 arbitrary units; furthermore, an automated full dependent MS-MS scan was applied. The injected samples were chromatographically separated in Thermo Instrument HPLC system (Accela PDA detector (Waldbronn, Germany), Accela PDA autosampler and Accela Pump, Agilent Technologies, Waldbronn, Germany) using a C18 (Sunfire 150 × 46 mm) column. The gradient elution for separation was CH_3_CN/H_2_O with 0.1% trifluoroacetic acid (TFA) (from 0% to 100% for 30 min, flow rate, 1.0 mL/min, UV detection max 340 nm).

1D and 2D NMR spectra were acquired on a Bruker AVANCE III HD 600 MHz (AscendTM14.1 Tesla, UK) with Prodigy TCI^TM^ cryoprobe at 298 K in CD_3_OD and DMSO-*d_6_* (Goss Scientific, Massachusetts, MA, USA). Trimethylsilane (TMS) was used as an internal standard. 

The optical rotation was measured using ADP 410 polarimeter (Bellingham + Stanley Ltd. 2007, Kent, UK) equipped with a light emitting diode and interference filter. A Fourier transform infrared (FTIR) spectrometer (2013, PerkinElmer, UK) equipped with an Attenuated Total Reflection (ATR, PerkinElmer, Buckinghamshire, UK) diamond cell for sample loading was used for infrared spectroscopy experiments.

### 3.2. Biological Material Collection and Identification 

The soil bacterium *Streptomyces* sp*. CT37* was isolated from the rhizosphere sample collected near the root of a *Caesalpinoideae* tree (Tamarindus Indica, Africa) growing in the Botanical Gardens of the University of Ghana, Legon (5°39′32.72” N, 0°11′55.26” W). The pure strain was cultured following the protocol given by the International Streptomyces Project (ISP) at 28 °C, supplemented with nalidixic acid and nystatin (25 mg/L) [22]. 

### 3.3. Smale Scale Cultivation of Streptomyces sp. CT37

The small scale culture (50 mL) of Streptomyces sp. CT37 was prepared by inoculating a single colony of the bacteria in eight different liquid culture media (ISP2, ISP3, ISP4, ISP5, ISP6, ISP7, modified Bennett’s, and starch casein) (Table S1), and incubated at 28 °C and 180 rpm for 7 days (Incu-shake FL16-2, SciQuip, Shrewsbury, UK). Subsequently, Diaion® HP-20 (3 g/50 mL, Mitsubishi Chemical Co., Binasco, Italy) was added to the fermentation cultures and incubated for the next 18–24 h at the same temperature and in the same shaking conditions. The culture broths were filtered under vacuum (Buchi pump V100, Buchi, Manchester, UK), and the HP-20 resin was rinsed with Milli-Q water and extracted exhaustively with methanol (MeOH, Fisher Chemical HPLC grade). All the methanol extracts were combined, and concentrated under reduced pressure (Buchi Rotavapor R200, Buchi, Manchester, UK) to generate the total crude extract (6.1 g). All eight crude extracts were then subjected to disc diffusion tests.

### 3.4. Disc Diffusion Assay

Disc diffusion assay was carried out following Kirby-Bauer disc diffusion susceptibility test Protocol [42]. Agar plates were inoculated with a standardized inoculum of pathogens (*Candida albicans* ATCC 10231, *Escherichia coli* ATCC 25922, *Pseudomonas aeruginosa* ATCC 27853, *Staphylococcus aureus* ATCC 25923, *Streptococcus* B. ATCC 12386, *Staphylococcus epidermidis ATCC 35984, Enterococcus faecalis ATCC 29212*, Table S2). Thereafter, filter paper discs (6 mm) impregnated with the test extracts (1 mg/mL) were placed on the agar surface. Oxytetracycline (30 µg/mL, Oxoid, Winchester, UK) was used as antibiotic control, while the growth media and Milli-Q water were used as negative control. The petri dishes were incubated at 37 °C for 18 h (Memmert INB200, Buchenbach, Germany), and the diameters of inhibition growth zones were measured. The ISP2 culture medium showed inhibition zone against *C. albicans;* hence it was scaled up. 

### 3.5. Large-Scale Fermentation of Streptomyces sp. CT37 

The seed culture of *Streptomyces* sp. CT37 (50 mL) was prepared following the same inoculation procedures as the small-scale cultivation. The seed culture was then used to inoculate (1:100) twenty-four 2.0-L baffled flasks (polycarbonate Corning^TM^, Flintshire, UK) containing 250 mL ISP2 broth each. The flasks were plugged with foam stoppers (polyurethane Fisherbrand™, Loughborough, UK). Fermentation, incubation, and extraction of the cultures were carried out as described above. The methanol extracts were combined, and evaporated to dryness under reduced pressure to yield 6.1 g of total crude extract, which was then fractionated using Strata® C18-E solid phase extraction (SPE) (55 µm, 70 Å, 20 g/60 mL) cartridges. The SPE column was equilibrated with four column volumes (CV) of methanol and Milli-Q water prior to loading the crude sample onto the column. The column was then eluted stepwise with solvent mixtures of decreasing polarity (8 CV/solvent mixture): Milli-Q water, 25% MeOH, 50% MeOH, 75% MeOH, 100% MeOH, and 100% MeOH with 0.05% TFA (Acros Organics, Morris, NJ, USA). The eluents were collected separately and labeled as fractions S1–S6. All the fractions (S1–S6) were concentrated under reduced pressure and subjected to HR-ESIMS analysis (0.1 mg/mL).

### 3.6. Feature Based Molecular Networking 

The MS/MS data of S1–S6 fractions were converted from .RAW to .mzXML files using the ProteoWizard MSconvert software [43]. A molecular network was generated using Feature-Based Molecular Networking (FBMN) workflow [44] on Global Natural Product Social networking (GNPS) [17] (https://gnps.ucsd.edu). The mass spectrometry data were preprocessed with MZMINE v2.38 [45] and exported to GNPS for FBMN analysis. The data were filtered to remove all MS/MS fragment ions within ±17 Da of the precursor *m/z*. MS/MS spectra were window filtered by choosing only the top 6 fragment ions in the ±50 Da window throughout the spectrum. The precursor ion mass tolerance was set to 0.02 Da with an MS/MS fragment ion tolerance of 0.02 Da to create consensus spectra. The consensus spectra that contained fewer than four spectra were discarded. The edges were filtered to ensure a cosine score above 0.65 and more than four matched peaks. The edges between two nodes were kept in the network if each of the nodes appeared in each other’s respective top 15 most similar nodes. The spectra in the network were then searched against GNPS spectral libraries [46] and annotated by the DEREPLICATOR [23]. The library spectra were filtered in the same manner as the input data, where a score above 0.65 and at least 4 matched peaks are required. The created molecular network was visualized using Cytoscape software v3.4.0 (Seattle, WA, US).

### 3.7. HPLC Isolation 

The compounds of interest were identified in S2 fraction, hence further fractionation was carried out in this fraction using High Pressure Liquid Chromatography (HPLC, Agilent Technologies 1260 infinity, Waldbronn, Germany). The purification was performed using a linear gradient from 10% H2O:MeOH (95:5) to 100% MeOH for 40 min with a solvent flow rate of 1.5 mL/min (C-18 ACE 10 µM 10 × 250 mm column). As a result, **1** (2.1 mg), **2** (1.1 mg), **3** (1.0 mg), and **4** (0.8 mg) were isolated.

*Legonimide***1**: pale yellow amorphous solid; UV (CH_3_OH): 250 nm; ^1^H, ^13^C NMR data, see Table 1; HR-ESIMS (+) *m/z* 293.1280 [M + H]^+^ (*calcd*. for C_18_H_17_N_2_O_2_^+^, 293.1285; ∆ = −1.70 ppm). 

^1^H-indole-3-carboxaldehyde **2**: pale yellow solid; UV(CH_3_OH) 240, 260, 280 nm; ^1^H, ^13^C NMR data, see Table S4; HR-ESIMS (+) [M + H]^+^ = 146.0603 (*calcd*. for C_9_H_8_NO^+^ 146.060, ∆ = 1.37 ppm).

*Actinopolymorphol B***3**: yellow solid; UV (MeOH) max nm 220, 280; [α]^25^ +27.3 (c = 0.5, CH_3_OH); ^1^H, ^13^C NMR data, see Table S4; HR-ESIMS (+) [M + H]^+^ = 204.1023 (calcd. for C_12_H_14_NO_2_^+^, 204.1019, ∆ = 1.96 ppm).

*(2R,3R)-1-phenylbutane-2,3-diol***4**. yellow oil; [α]^25^_D_ +12.1 (c 0.5, MeOH); UV (MeOH) max nm 250; IR νmax (cm^−1^) 3410, 2931, 2874, 1712, 1645, 1409, 1207, 1012, 802, 754; ^1^H, ^13^C NMR data, see Table S5; HR-ESIMS (+) [M + H]^+^ = 167.1070 (calcd. for C_10_H_15_O_2_^+^, 167.1067, ∆ = −1.80 ppm).

### 3.8. Minimum Inhibitory Concentration 

Minimum inhibitory concentrations (MIC) of **1**–**4** against *C. albicans* ATCC 10,231 were determined using a conventional broth dilution assay in accordance with standards recommended by the National Committee for Clinical Laboratory Standards (NCCLS) [47] and as described previously [7]. The antibiotics ampicillin (Sigma) and tetracycline (Sigma) were used as standards. The absorbance was recorded after 24 h (OD_600_) in a SpectraMax ABS Plus (Molecular Device) plate reader. The MIC was defined as the lowest concentration of compound that inhibited ≥ 95% of the growth of *C. albicans* after overnight incubation.

## 4. Conclusions

The bioassay and molecular network-assisted isolation afforded four alkaloids **1-4** from the Ghanaian soil bacterium, *Streptomyces* sp. CT37. Their structures were deduced by analysis of the HRESIMS, 1D, and 2D NMR. Legonimide **1** was identified as a new alkaloid bearing the imide motif in its structure, while **2**-**4** were known and their structures were elucidated as 1H-indole-3-carbaldehyde, 3-hydroxy-4-(1H-indol-3-yl) butan-2-one, and (2*R*,3*R*)-1-phenylbutane-2,3-diol, respectively. Alkaloids **1** and **2** exhibited moderate inhibition against *C. albicans* ATCC 10,231 with MIC values of 21.54 µg/mL and 11.47 µg/mL, respectively. 

## Figures and Tables

**Table 1:** ^1^H and ^13^C NMR assignments of legonimide **1** (600MHz, 298K, DMSO-*d_6_*).

Position	^1^H (mult., *J* in Hz)	^13^C
1-NH	10.96 (1H, s)	-
2	7.18 (1H, s)	123.6
3	-	109.2
4a	-	127.3
4	7.54 (1H, d, *J* = 7.85)	118.7
5	7.02 (1H, t, *J* = 7.39)	118.5
6	7.06 (1H, t, *J* = 7.32)	120.8
7	7.34 (1H, d, *J* = 7.80)	111.3
7a	-	136.5
8	3.46(2H, s)	32.4
9	-	173.1
10-NH	7.37 (1H, s)	-
11	-	172.3
12	3.35(2H, s)	42.0
13	-	136.6
14,14′	7.28 (2H, d, *J* = 7.67)	128.1
15,15′	7.21 (2H, m)	126.4
16	7.31 (1H, m)	126.3

**Table 2:** Minimum inhibitory concentration of compounds **1**–**4** against *C. albicans* 10231.

	*C. albicans* ATCC 10231 MIC (µg/mL)
Legonimide **1**	21.54
Compound **2**	11.47
Compound **3**	>50
Compound **4**	>50
Ampicillin	3.193
Tetracycline	0.3615

## Supplementary Materials

The supplementary materials are available online.

## References

[1] Ngo L.T., Okogun J.I., Folk W.R. (2013). 21st Century natural product research and drug development and traditional medicines. Nat. Prod. Rep..

[2] Mohana N.C., Rao H.C.Y., Rakshith D., Mithun P.R., Nuthan B.R., Satish S. (2018). Omics based approach for biodiscovery of microbial natural products in antibiotic resistance era. J. Genet. Eng. Biotechnol..

[3] Hopwood D.A. (2007). How do antibiotic-producing bacteria ensure their self-resistance before antibiotic biosynthesis incapacitates them?. Mol. Microbiol..

[4] Fang Q., Maglangit F., Wu L., Ebel R., Kyeremeh K., Andersen J.H., Annang F., Pérez-Moreno G., Reyes F., Deng H. (2020). Signalling and bioactive metabolites from *Streptomyces* sp. RK44. Molecules.

[5] Deng H., Ma L., Bandaranayaka N., Qin Z., Mann G., Kyeremeh K., Yu Y., Shepherd T., Naismith J.H., O’Hagan D. (2014). Identification of Fluorinases from *Streptomyces* sp MA37, *Norcardia brasiliensis*, and *Actinoplanes* sp N902-109 by genome mining. ChemBioChem.

[6] Maglangit F., Tong M.H., Jaspars M., Kyeremeh K., Deng H. (2019). Legonoxamines A-B, two new hydroxamate siderophores from the soil bacterium, *Streptomyces* sp. MA37. Tetrahedron Lett..

[7] Maglangit F., Fang Q., Leman V., Soldatou S., Ebel R., Kyeremeh K., Deng H. (2019). Accramycin A, a new aromatic polyketide, from the soil bacterium, *Streptomyces* sp. MA37. Molecules.

[8] Marmann A., Aly A.H., Lin W., Wang B., Proksch P. (2014). Co-cultivation—A powerful emerging tool for enhancing the chemical diversity of microorganisms. Mar. Drugs.

[9] Tabudravu J.N., Pellissier L., Smith A.J., Subko K., Autreáu C., Feussner K., Hardy D., Butler D., Kidd R., Milton E.J. (2019). LC-HRMS-database screening metrics for rapid prioritization of samples to accelerate the discovery of structurally new natural products. J. Nat. Prod..

[10] Su L., Zhang R., Kyeremeh K., Deng Z., Deng H., Yu Y. (2017). Dissection of the neocarazostatin: A C_4_ alkyl side chain biosynthesis by in vitro reconstitution. Org. Biomol. Chem..

[11] Su L., Lv M., Kyeremeh K., Deng Z., Deng H., Yu Y. (2016). A ThDP-dependent enzymatic carboligation reaction involved in neocarazostatin a tricyclic carbazole formation. Org. Biomol. Chem..

[12] Rateb M.E., Zhai Y., Ehrner E., Rath C.M., Wang X., Tabudravu J., Ebel R., Bibb M., Kyeremeh K., Dorrestein P.C. (2015). Legonaridin, a new member of linaridin RiPP from a Ghanaian Streptomyces isolate. Org. Biomol. Chem..

[13] Huang S., Tabudravu J., Elsayed S.S., Travert J., Peace D., Tong M.H., Kyeremeh K., Kelly S.M., Trembleau L., Ebel R. (2015). Discovery of a single monooxygenase that catalyzes carbamate formation and ring contraction in the biosynthesis of the legonmycins. Angew. Chem. Int. Ed..

[14] Maglangit F., Fang Q., Kyeremeh K., Sternberg J.M., Ebel R., Deng H. (2020). Co-Culturing Approach Enables Discovery and Biosynthesis of a Bioactive Indole Alkaloid Metabolite. Molecules.

[15] Callahan D.L., Elliott C.E., Walker J.M. (2013). Metabolomics Tools for Natural Product Discovery.

[16] Cox D.G., Oh J., Keasling A., Colson K., Hamann M.T. (2014). The utility of metabolomics in natural product and biomarker characterization. Biochim. Biophys. Acta.

[17] Networking S.M., Wang M., Carver J.J., Phelan V.V., Sanchez L.M., Garg N., Peng Y., Nguyen D.D.D.T., Watrous J., Kapono C.A. (2016). Sharing and community curation of mass spectrometry data with global natural products social molecular networking. Nat. Biotechnol..

[18] Ernst M., Kang K.B., Caraballo-Rodríguez A.M., Nothias L.-F., Wandy J., Chen C., Wang M., Rogers S., Medema M.H., Dorrestein P.C. (2019). MolNetEnhancer: Enhanced molecular networks by integrating metabolome mining and annotation tools. Metabolites.

[19] Borges R.M., Taujale R., de Souza J.S., de Andrade Bezerra T., Silva E.L.E., Herzog R., Ponce F.V., Wolfender J.-L., Edison A.S. (2018). Dereplication of plant phenolics using a mass-spectrometry database independent method. Phytochem. Anal..

[20] Blaženović I., Kind T., Torbašinović H., Obrenović S., Mehta S.S., Tsugawa H., Wermuth T., Schauer N., Jahn M., Biedendieck R. (2017). Comprehensive comparison of in silico MS/MS fragmentation tools of the CASMI contest: Database boosting is needed to achieve 93% accuracy. J. Cheminform..

[21] Bode H.B., Bethe B., Höfs R., Zeeck A. (2002). Big effects from small changes: Possible ways to explore nature’s chemical diversity. ChemBioChem.

[22] Shirling E.B., Gottlieb D. (1966). Methods for characterization of streptomyces species. Int. J. Syst. Bacteriol..

[23] Mohimani H., Gurevich A., Shlemov A., Mikheenko A., Korobeynikov A., Cao L., Shcherbin E., Nothias L.-F., Dorrestein P.C., Pevzner P.A. (2018). Dereplication of microbial metabolites through database search of mass spectra. Nat. Commun..

[24] Santen J., Van A., Jacob G., Singh A.L., Aniebok V., Balunas M.J., Bunsko D., Neto F.C., Castaño-Espriu L., Chang C. (2019). The natural products atlas: An open access knowledge base for microbial natural products discovery. ACS Cent. Sci..

[25] Sun D., Dong W., Li X., Zhang H. (2010). Indole alkaloids from the roots of *isatis ingigotica* and their antiherpes simplex virus type 2 (HSV-2) activity *in vitro*. Chem. Nat. Compd..

[26] Awano K.I., Yanai T., Watanabe I., Takagi Y., Kitahara T., Mori K. (1995). Synthesis of all four possible stereoisomers of 1-phenyl-2,3-butanediol and both enantiomers of 3-hydroxy-4-phenyl-2-butanone to determine the absolute configuration of the natural constituents. Biosci. Biotechnol. Biochem..

[27] Huang S.X., Powell E., Rajski S.R., Zhao L.X., Jiang C.L., Duan Y., Xu W., Shen B. (2010). Discovery and total synthesis of a new estrogen receptor heterodimerizing actinopolymorphol A from actinopolymorpha rutilus. Org. Lett..

[28] Parra R.D., Furukawa M., Gong B., Zeng X.C. (2001). Energetics and cooperativity in three-center hydrogen bonding interactions. I. diacetamide-X dimers (X=HCN, CH_3_OH). J. Chem. Phys..

[29] Hvoslef J., Tracy M.L., Nash C.P. (1986). Interatomic distances and angles in four planar systems with adjacent C–O and C–N bonds: Structures of pivalamide (I), dipivalamide (II),N-pivaloylpivalamidinium pyrosulfate (III) and N-pivaloylpivalamidine (IV). Acta Crystallogr. Sect. C Cryst. Struct. Commun..

[30] Hinterberger S., Hofer O., Greger H. (1994). Synthesis and corrected structures of sulphur-containing amides from glycosmis species: Sinharines, penimides, and illukumbins. Tetrahedron.

[31] Cardoso-Martínez F., De La Rosa J.M., Díaz-Marrero A.R., Darias J., D’Croz L., Cerella C., Diederich M., Cueto M. (2015). Oximoaspergillimide, a fungal derivative from a marine isolate of *Aspergillus* sp.. Eur. J. Org. Chem..

[32] Karplus M. (1959). Contact electron-spin coupling of nuclear magnetic moments. J. Chem. Phys..

[33] Yu L., Liu J., Yu L., Chen L., Qiu F. (2018). Chemical constituents of seed oil leavings of xanthoceras sorbifolia. Chem. Nat. Compd..

[34] Netz N., Opatz T. (2015). Marine indole alkaloids. Mar. Drugs.

[35] Yang S.W., Cordell G.A. (1997). Metabolism studies of indole derivatives using a staurosporine producer, Streptomyces staurosporeus. J. Nat. Prod..

[36] Lacret R., Oves-Costales D., Gómez C., Díaz C., De La Cruz M., Pérez-Victoria I., Vicente F., Genilloud O., Reyes F. (2015). New ikarugamycin derivatives with antifungal and antibacterial properties from streptomyces zhaozhouensis. Mar. Drugs.

[37] Cartus A.T., Stegmüller S., Simson N., Wahl A., Neef S., Kelm H., Schrenk D. (2015). Hepatic metabolism of carcinogenic β-asarone. Chem. Res. Toxicol..

[38] Xie C.L., Niu S.W., Zhou T.T., Zhang G.Y., Yang Q., Yang X.W. (2016). Chemical constituents and chemotaxonomic study on the marine actinomycete *Williamsia* sp. MCCC 1A11233. Biochem. Syst. Ecol..

[39] Böttcher C., Chapman A., Fellermeier F., Choudhary M., Scheel D., Glawischnig E. (2014). The biosynthetic pathway of indole-3-carbaldehyde and indole-3-carboxylic acid derivatives in arabidopsis. Plant Physiol..

[40] Khan A.R., Park G.S., Asaf S., Hong S.J., Jung B.K., Shin J.H. (2017). Complete genome analysis of Serratia marcescens RSC-14: A plant growth-promoting bacterium that alleviates cadmium stress in host plants. PLoS ONE.

[41] Zhang L.S., Davies S.S. (2016). Microbial metabolism of dietary components to bioactive metabolites: Opportunities for new therapeutic interventions. Genome Med..

[42] Hudzicki J. (2009). Kirby-bauer disk diffusion susceptibility test protocol author information. Am. Soc. Microbiol..

[43] Kessner D., Chambers M., Burke R., Agus D., Mallick P. (2008). ProteoWizard: Open source software for rapid proteomics tools development. Bioinformatics.

[44] Nothias L.F., Petras D., Schmid R., Dührkop K., Rainer J., Sarvepalli A., Protsyuk I., Ernst M., Tsugawa H. (2019). Feature-based molecular networking in the GNPS analysis environment. BiorXiv.

[45] Pluskal T., Castillo S., Villar-briones A., Orešič M., Ore M. (2010). MZmine 2: Modular framework for processing, visualizing, and analyzing mass spectrometry-based molecular profile data. BMC Bioinform..

[46] Horai H., Arita M., Kanaya S., Nihei Y., Ikeda T., Suwa K., Ojima Y., Tanaka K., Tanaka S., Aoshima K. (2010). MassBank: A public repository for sharing mass spectral data for life sciences. J. Mass Spectrom..

[47] Carpenter D.E., Karen Anderson Diane Citron C.M., JoAnn Dzink-Fox B.L., Meredith Hackel M., Jenkins S.G., Cindy Knapp F., Laura Koeth M., Audrey Schuetz M.N., Hannah Wexler D. (2018). Methods for Antimicrobial Susceptibility Testing of Anaerobic Bacteria.

