# Supplementary material for: Targeted Isolation of Indole Alkaloids from Streptomyces sp. CT37
Source: Molecules. 2020 Mar 2;25(5):1108. doi: 10.3390/molecules25051108 (PMC7179168; doi:10.3390/molecules25051108)
Supplement: Supplementary file 1 [file molecules-25-01108-s001.pdf]

Supporting Information

Targeted isolation of indole alkaloids from *Streptomyces* sp. CT37

Qing Fang, Fleurdeliz Maglangit, Morgane Mugat, Caroline Urwald, Kwaku Kyeremeh, Hai Deng

Contents:

Table S1. Culture media used in the study
Table S2. Disk diffusion antimicrobial test results of ISP2-ISP7, MB and SC extracts
Figure S1. Inhibition zones of ISP2-ISP7, MB and SC extracts against *C. albicans* ATCC 10231
Figure S2. Molecular network of *Streptomyces* sp. CT37 ISP2 extract
Table S3. MS^1 table for manual dereplication
Figure S3. Substructures A-D of legonimide **1**
Figure S4. ^{1}H-NMR of legonimide **1**
Figure S5. ^{1}H-NMR alignment of legonimide **1** in CD_3OD and DMSO-d_6
Figure S6. ^{13}C-NMR of legonimide **1**
Figure S7. HSQC of legonimide **1**
Figure S8. COSY of legonimide **1**
Figure S9-11. HMBC of legonimide **1**
Figure S12. imide conformers and their corresponding HMBC correlations
Figure S13. HMBC correlations of legonimide **1** showing *cis-trans* imide conformation
Table S4. ^{1}H-NMR and ^{13}C-NMR comparison of **2-3** with reported metabolites in literature
Figure S14. ^{1}H-NMR of **2**
Figure S15. HSQC of **2**
Figure S16. COSY of **2**
Figure S17. HMBC of **2**
Figure S18. ^{13}C-NMR of **2**
Figure S19. ^{1}H-NMR of **3**
Figure S20. HSQC of **3**
Figure S21. COSY of **3**
Figure S22. HMBC of **3**
Figure S23. ^{13}C-NMR of **3**
Figure S24. ^{1}H-NMR of **4**
Figure S25. HSQC of **4**
Figure S26. COSY of **4**
Figure S27. HMBC of **4**
Figure S28. ^{13}C-NMR of **4**
Table S5. ^{1}H-NMR and ^{13}C-NMR comparison of **4** with reported metabolite in literature
Table S6. Optical rotation (OR) of **4** in comparison with the reported OR of **4** stereoisomers
Figure S29. MIC curve of **1** and **2** against *Candida albicans* ATCC 102317

References

Table S1. Culture media used in the study

Media	Composition
Modified Bennett's (MB)	Glycerol 10g, Bacto-Casitone 2g, Yeast Extract 1g, Lab-Lemco 0.8g
ISP2	Glucose 4 g, Yeast extract 4 g, Malt extract 10 g, milliQ water 1L
ISP3	pH = 7.2, Oatmeal 20 g, milliQ water 1L<br>Trace elements: 1 mL ($FeSO_4.7H_2O$ 0.1g, $MnCl_2.4H_2O$ 0.1g, $ZnSO_4.7H_2O$, 0.1g per liter)
ISP4	pH 7.2<br>Solution 1: 500 mL, Difco soluble starch 10.0 g/500 mL water<br>Solution 2: 500 mL, K_2HPO_4 1 g, $MgSO_4 \cdot 7H_2O$ 1g, NaCl 1g, $(NH_4)_2SO_4$ 1g, $CaCO_3$ 1g, Trace elements (above) 1 mL, milliQ water 1L
ISP5	pH = 7.2<br>L-asparagine (anhydrous) 1.0g, glycerol 10.0 g, K_2HPO_4 1.0 g (anhydrous), trace salts solution (above) 1.0 mL, milliQ water 1L
ISP6	Bacto-peptone 15 g, proteose peptone (Difco) 5 g, ferric ammonium citrate 0.5 g, dipotassium phosphate 1 g, sodium thiosulfate 0.08 g, Bacto-Yeast extract (Difco) 1 g, milliQ water 1L
ISP7	pH 7.2<br>Glycerol 15g, L-tyrosine 0.5 g, L- asparagine (Difco) 1 g, K_2HPO_4 (anhydrous) 0.5g, $MgSO_4 \cdot 7H_2O$, 0.5g NaCl, 0.5g $FeSO_4.7H_2O$, 0.01g Trace salts solution (above) 1 mL, milliQ water 1L
Starch Casein media (SC)	Starch 10g, casein 0.3g, KNO_3 2g, NaCl 2g, K_2HPO_4 2g, $MgSO_4 \cdot 7H_2O$ 5mg, $CaCO_3$ 2mg, $FeSO_4 \cdot 7H_2O$ 10mg

Table S2. Disc diffusion test results (in triplicates) of ISP2-ISP7, modified Bennett's (MB) and Starch Casein (SC) extracts against a panel of pathogens

			Average Inhibition zone (mm)							
	Pathogen	OT30*	ISP2	ISP3	ISP4	ISP5	ISP6	ISP7	MB	SC
Fungi	*Candida albicans* ATCC 10231	37	9.0	0	0	0	0	0	0	0
Gram-negative	*E. coli* ATCC 25922	18	0	0	0	0	0	0	0	0
	P. aeruginosa ATCC 27853	10	0	0	0	0	0	0	0	0
Gram-positive	*S. aureus* ATCC 25923	19	0	0	0	0	0	0	0	0
	Streptococcus B. ATCC 12386	19	0	0	0	0	0	0	0	0
	S. epidermidis ATCC 35984	22	0	0	0	0	0	0	0	0
	E. faecalis ATCC 29212	15	0	0	0	0	0	0	0	0

*OT30 – oxytetracyline antibiotic (30µg/mL, Sigma)

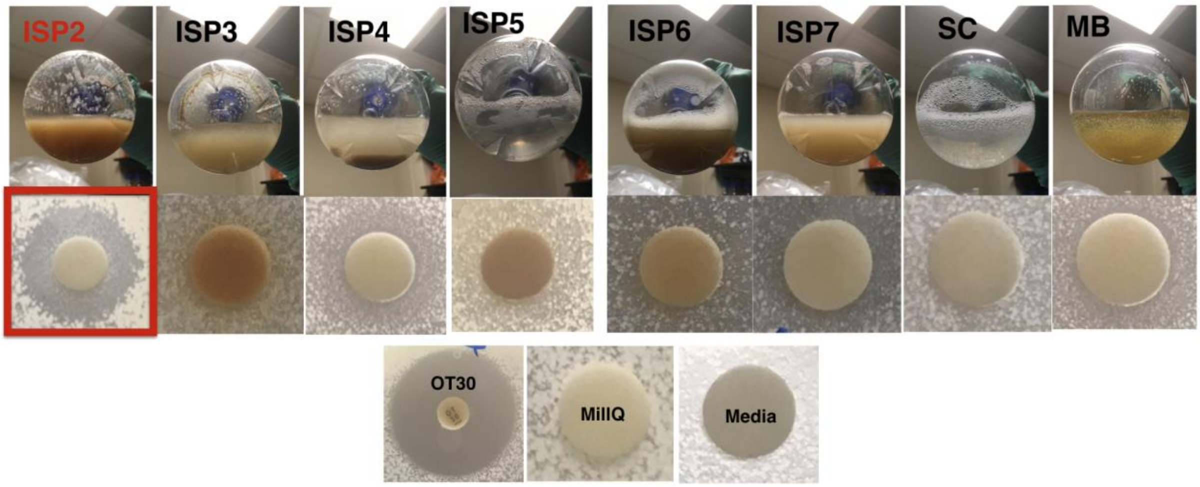

Figure S1. Zones of inhibition (in triplicates) of ISP2-ISP7, modified Bennett's (MB) and Starch Casein (SC) extracts against *Candida albicans* ATCC 10231. Diameter of inhibition zone was measured after 18hrs. (Positive control: OT30 – oxytetracyline antibiotic (30µg/mL, Sigma); negative control: Milli-Q water and medi

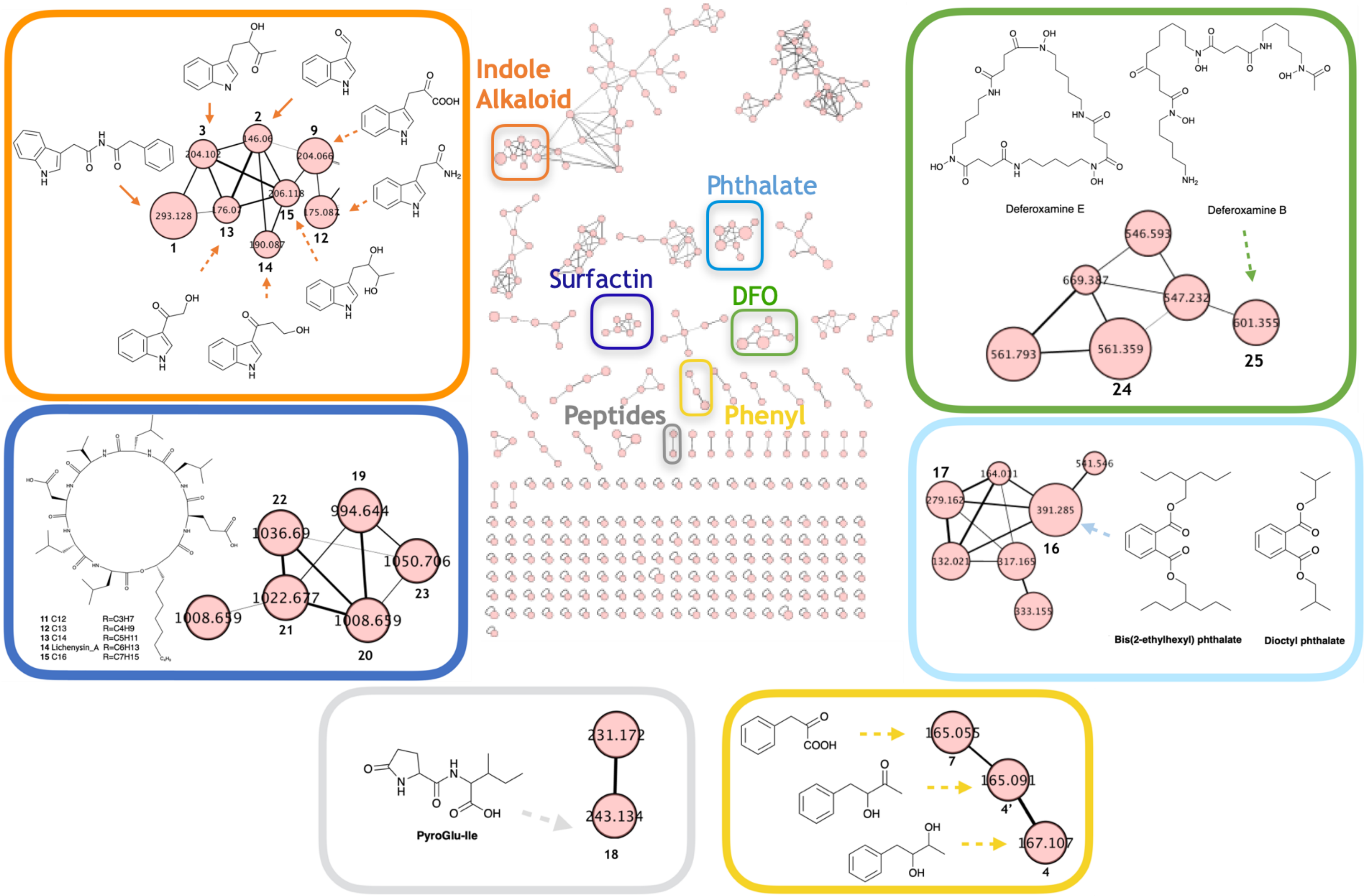

Figure S2. Molecular network of *Streptomyces* sp. CT37 showing clusters corresponding to indole alkaloids,phenyls, phthalates, surfactin, peptides, deferoxamines (DFO). The size of nodes represents the sum of ion intensity. The width of edge indicates the cosine score.

Table S3 MS^1 table for manual dereplication

ID	Obs(M+H$^+$)	Rt(min)	MN cluster	MF	Δppm	Dereplicated Compounds	Isolated compounds (NMR identification)
1	293.128	6.27	Indole Alkaloid	$C_{18}H_{16}N_2O_2$	-1.70	No Hit [c]	N-(2-(1H-indol-3-yl)acetyl)-2-phenylacetamide
2	146.060	4.05	Indole Alkaloid	C_9H_7NO	1.37	1H-indole-3-carbaldehyde [b]	1H-indole-3-carbaldehyde
3	204.102	4.65	Indole Alkaloid	$C_{12}H_{13}NO_2$	1.96	3-hydroxy-4-(1H-indol-3-yl)butan-2-one [b]	3-hydroxy-4-(1H-indol-3-yl)butan-2-one
9	204.066	4.59	Indole Alkaloid	$C_{11}H_9NO_3$	2.45	Indole-3-pyruvate [b]	ND
12	175.087	4.73	Indole Alkaloid	$C_{10}H_{10}N_2O$	2.28	Indole-3-acetamide [b]	ND
13	176.071	4.47	Indole Alkaloid	$C_{10}H_9NO_2$	2.27	2-hydroxy-1-(1H-indol-3-yl)ethenone [b]	ND
14	190.087	4.81	Indole Alkaloid	$C_{11}H_{11}NO_2$	1.05	3-hydroxy-1-(1H-indol-3-yl)propan-1-one [b]	ND
15	206.118	4.61	Indole Alkaloid	$C_{12}H_{15}NO_2$	1.94	1-(1H-indol-3-yl)butane-2,3-diol [b]	ND
4	167.107	4.13	Phenyl	$C_{10}H_{14}O_2$	-1.80	1-phenylbutane-2,3-diol [b]	(2R,3R)-1-phenylbutane-2,3-diol
4’	165.091	4.32	Phenyl	$C_{10}H_{12}O_2$	1.79	3-hydroxy-4-phenyl-2-butanone [b]	ND
7	165.055	4.01	Phenyl	$C_9H_8O_3$	2.43	phenylpyruvate [b]	ND
16	391.285	12.90	Phthalate	$C_{24}H_{38}O_4$	0.77	bis(2-ethylhexyl) phthalate [a]	ND
17	279.160	9.48	Phthalate	$C_{16}H_{22}O_4$	1.43	Dioctyl phthalate [a]	ND
18	243.134	4.68	Peptide	$C_{11}H_{18}N_2O_4$	1.23	PyroGlu-ile [a]	ND
19	994.644	9.81	Surfactin	$C_{50}H_{87}N_7O_{13}$	1.10	Surfactin C12 [a]	ND
20	1008.659	10.73	Surfactin	$C_{51}H_{89}N_7O_{13}$	1.39	Surfactin C13 [a]	ND
21	1022.677	11.65	Surfactin	$C_{52}H_{91}N_7O_{13}$	3.42	Surfactin C14 [a]	ND
22	1036.690	12.19	Surfactin	$C_{53}H_{93}N_7O_{13}$	0.10	Lichenysin A [a]	ND
23	1050.706	14.44	Surfactin	$C_{54}H_{95}N_7O_{13}$	3.71	Surfactin C16 [a]	ND
24	561.359	4.89	Deferoxamine	$C_{25}H_{48}N_6O_8$	-2.85	deferoxamine B [a]	ND
25	601.355	4.69	Deferoxamine	$C_{27}H_{48}N_6O_9$	-0.66	deferoxamine E [a]	ND

[a] Annotated compound from GNPS spectral libraries, [b] Annotated compound from manual dereplication, [c] No matching in GNPS libraries and manual dereplication

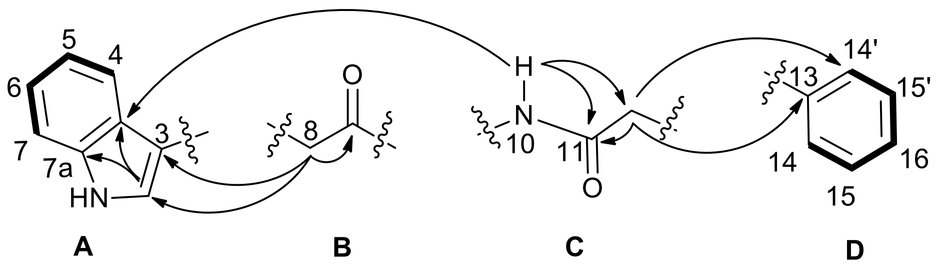

Figure S3. Substructures A-D of legonimide **1** with COSY (—) and key HMBC (→

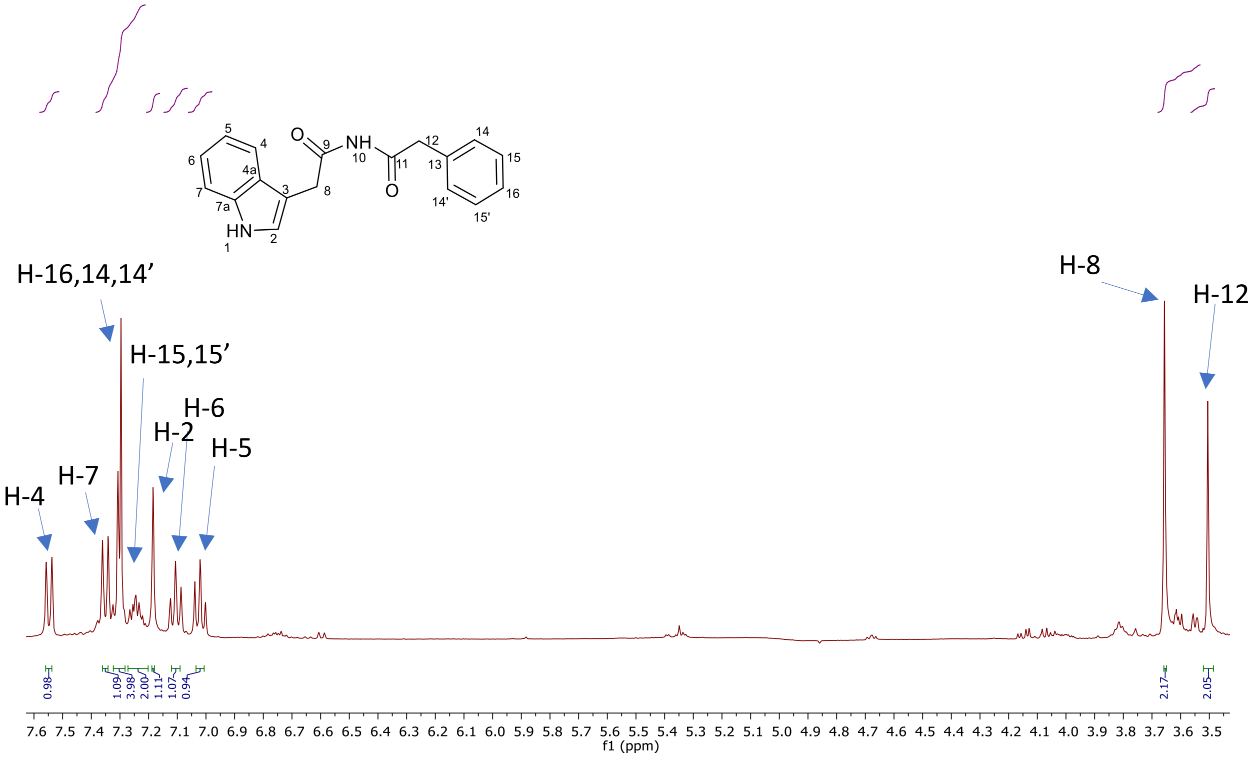

Figure S4. ^{1}H-NMR of legonimide **1** in CD_3OD at 600MHz

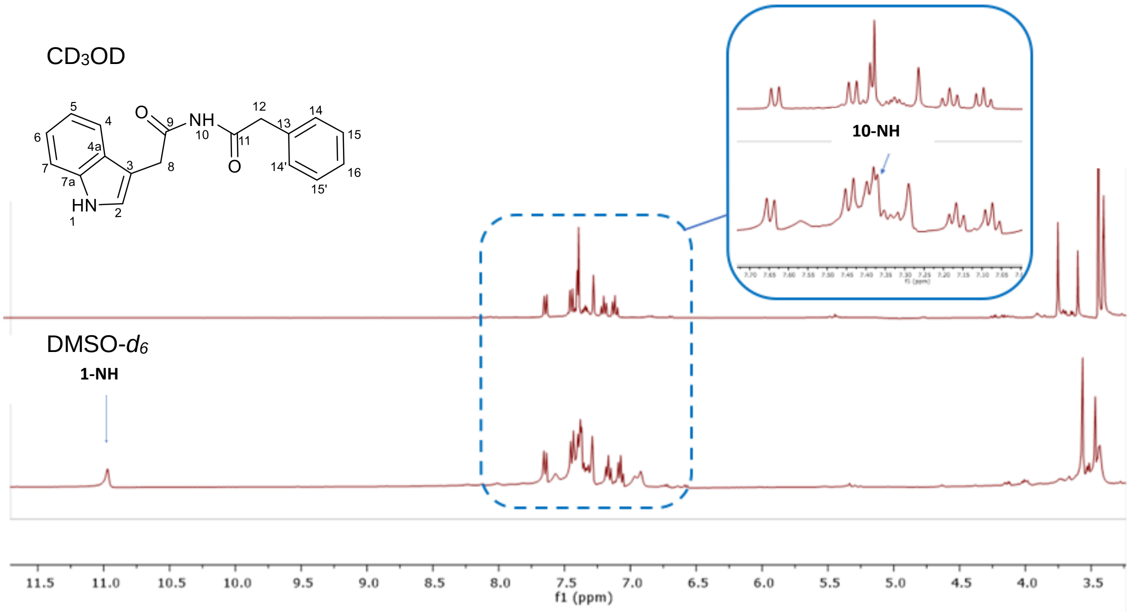

Figure S5. ^{1}H-NMR alignment of the NH groups in legonimide **1** in CD_3OD and DMSO-d_6

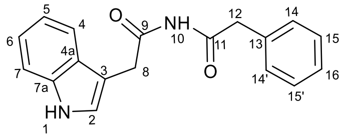

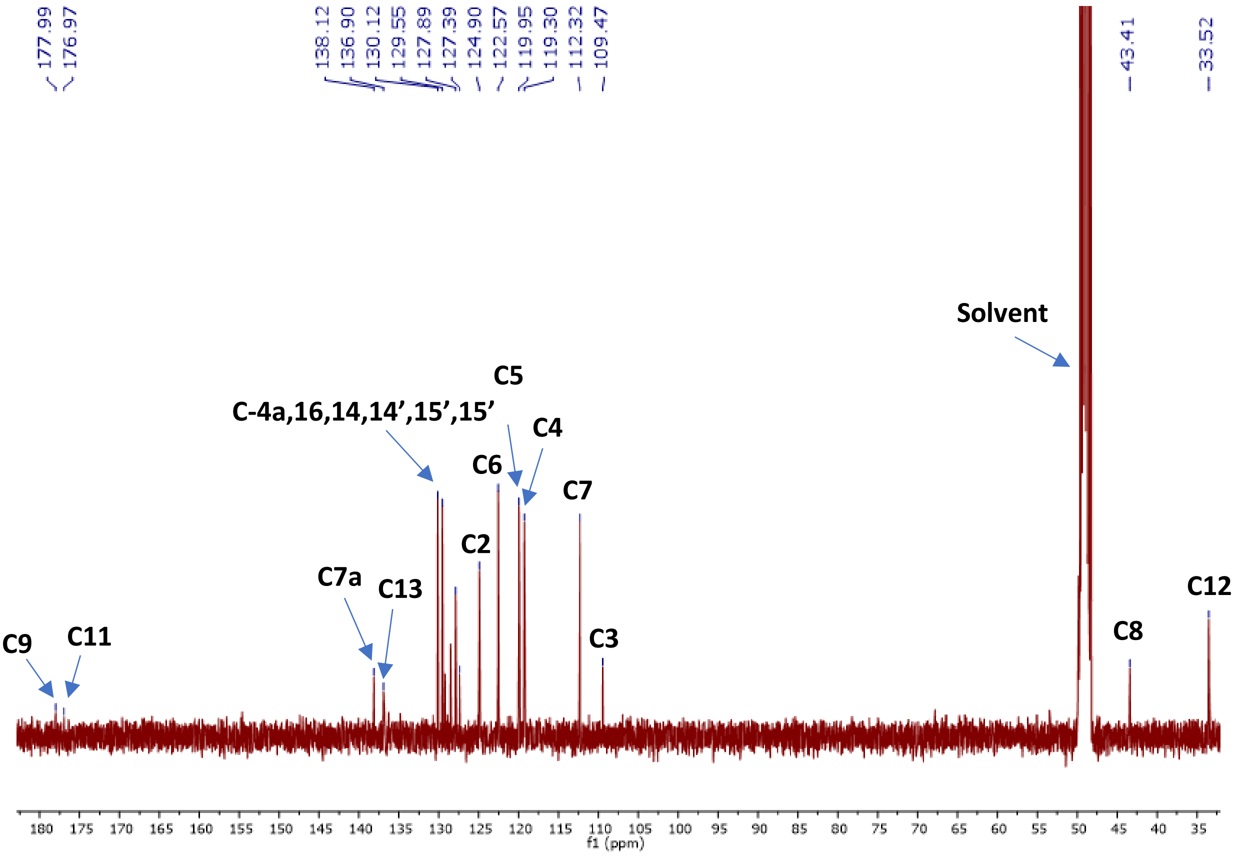

Figure S6. ^{13}C-NMR of legonimide **1** in CD_3OD at 600MHz

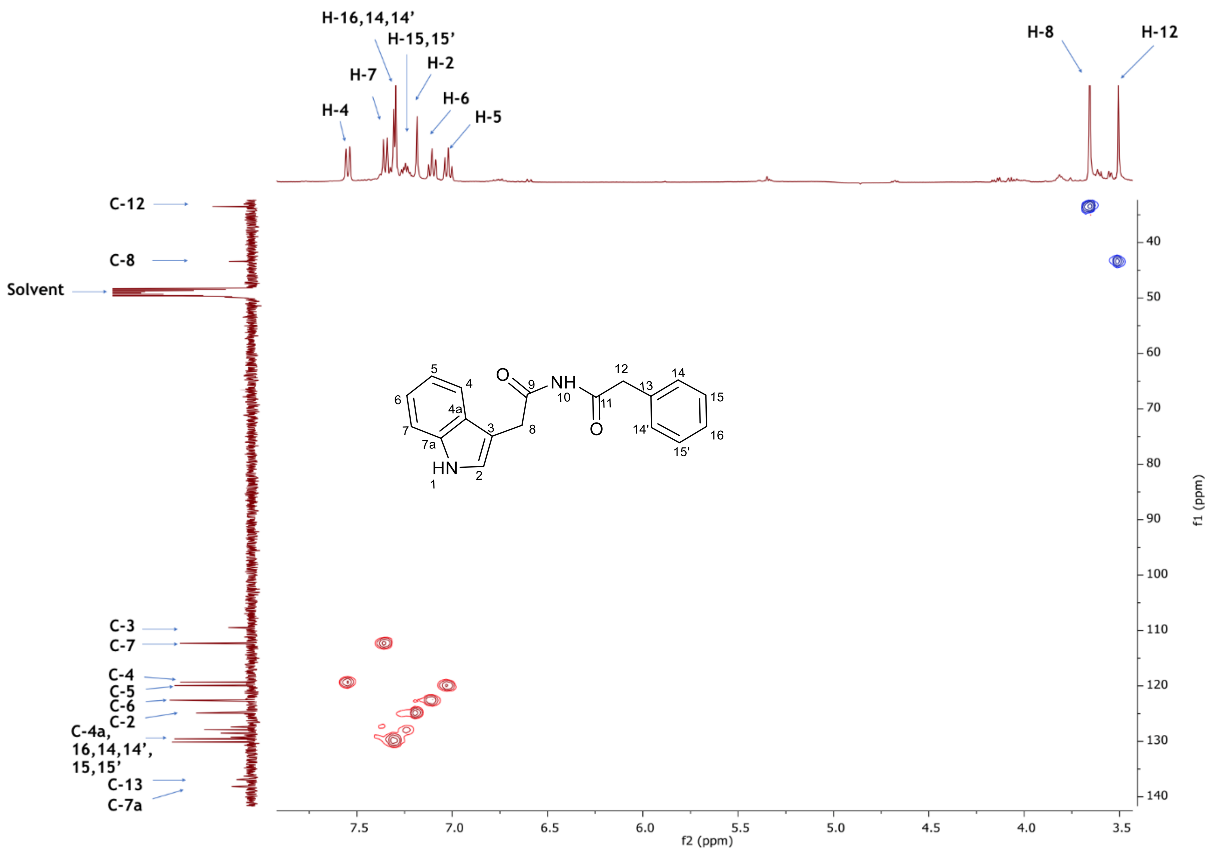

Figure S7. HSQC of legonimide **1** in CD_3OD at 600MHz

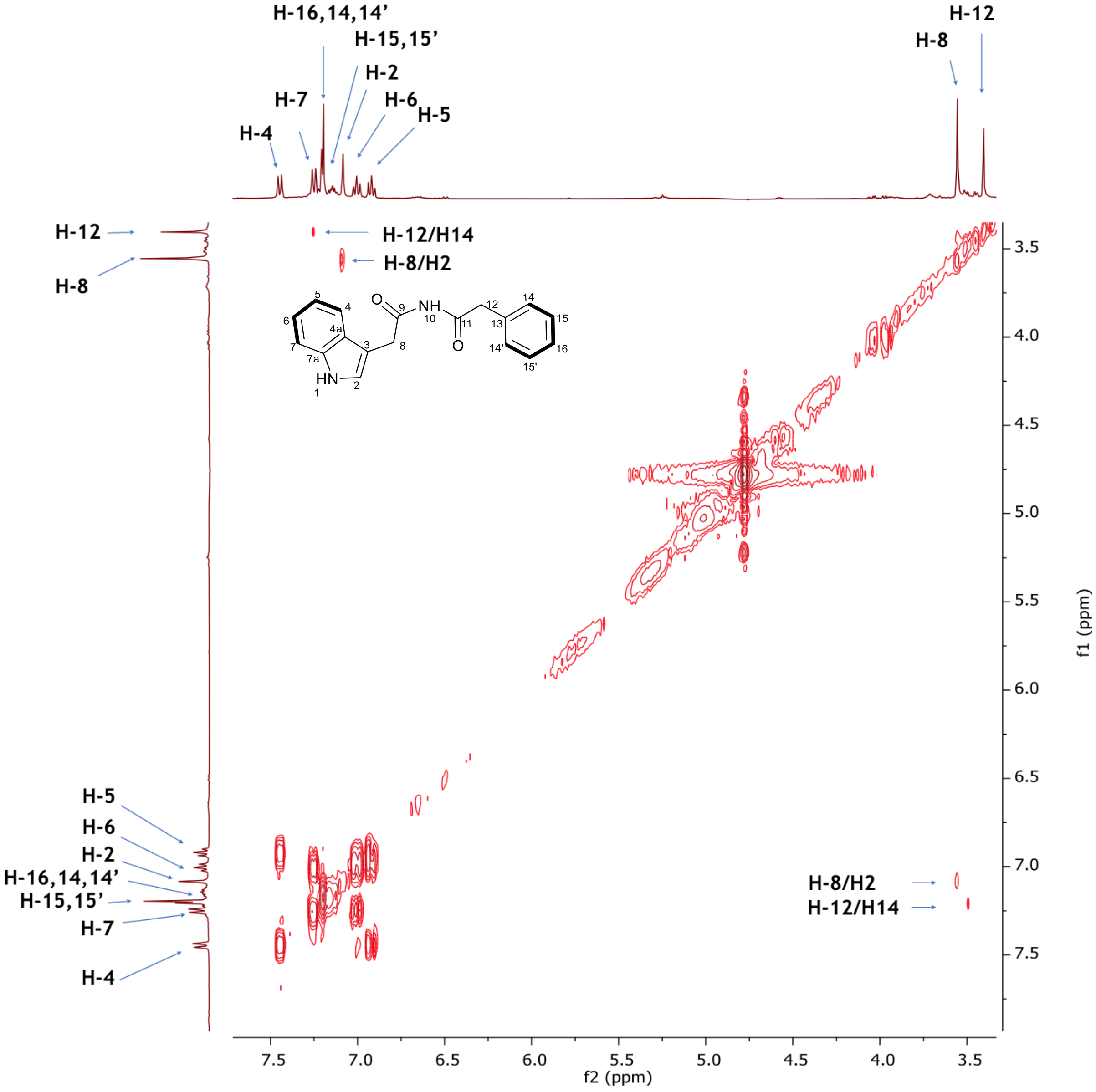

Figure S8. COSY of legonimide **1** in CD_3OD at 600MHz

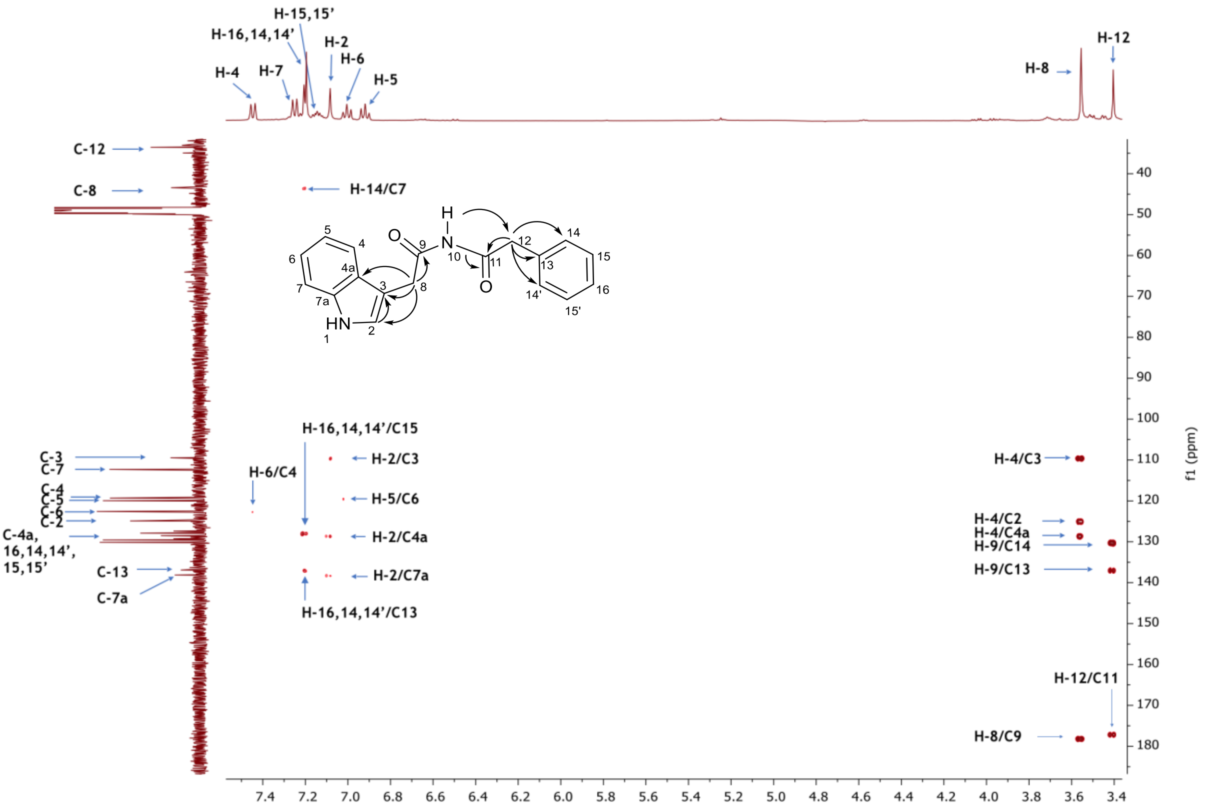

Figure S9. HMBC of legonimide **1** in CD_3OD at 600MHz

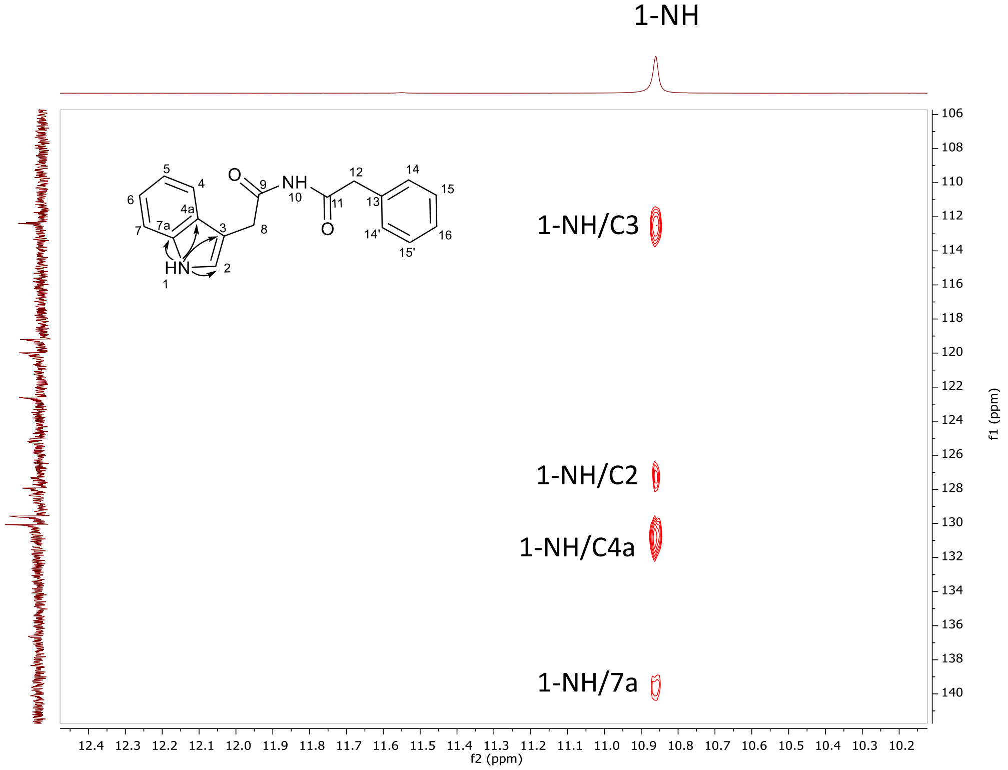

Figure S10. HMBC spectra of legonimide **1** showing correlations from NH-1 to C-2, C-3, C-4a, and C-7a (DMSO-d_6)

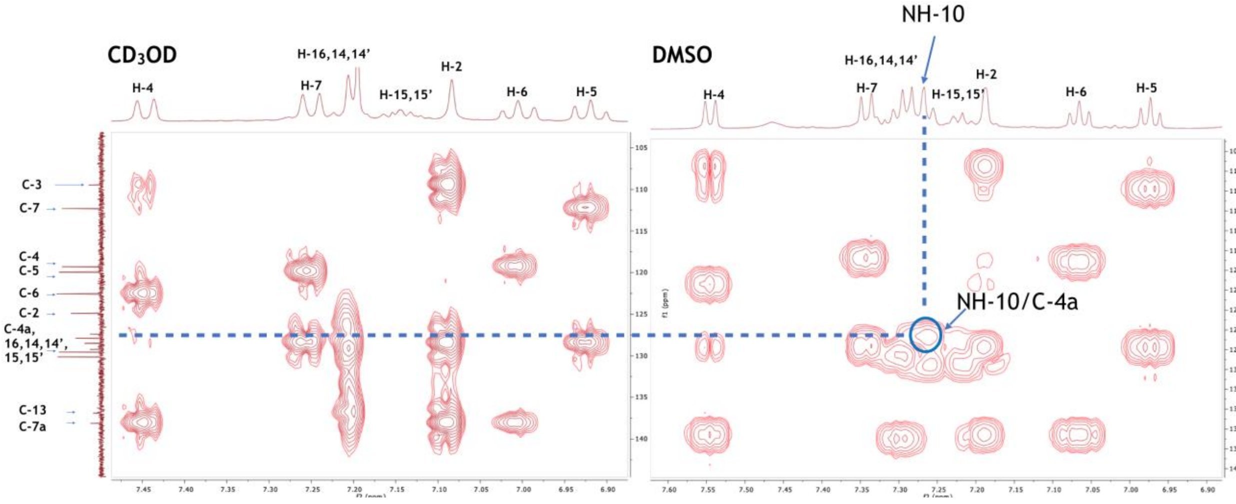

Figure S11. Comparison of the HMBC spectra of legonimide **1** in CD_3OD and DMSO-d_6. The DMSO-d_6 spectrum showed long-range correlation from NH-10 to C-4a

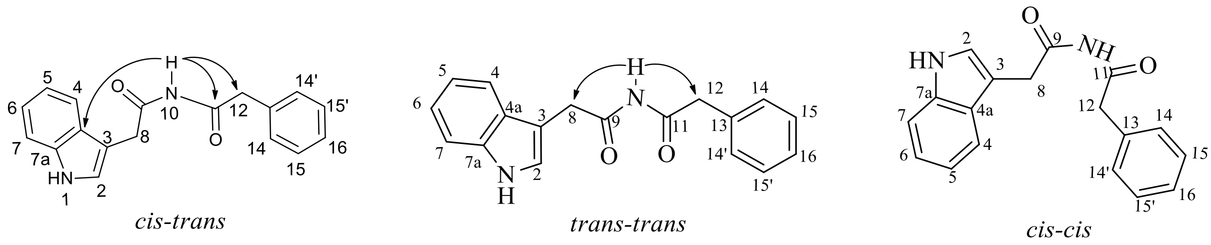

Figure S12. Imide (CONHCO) conformers and their corresponding HMBC correlations (→) [1]

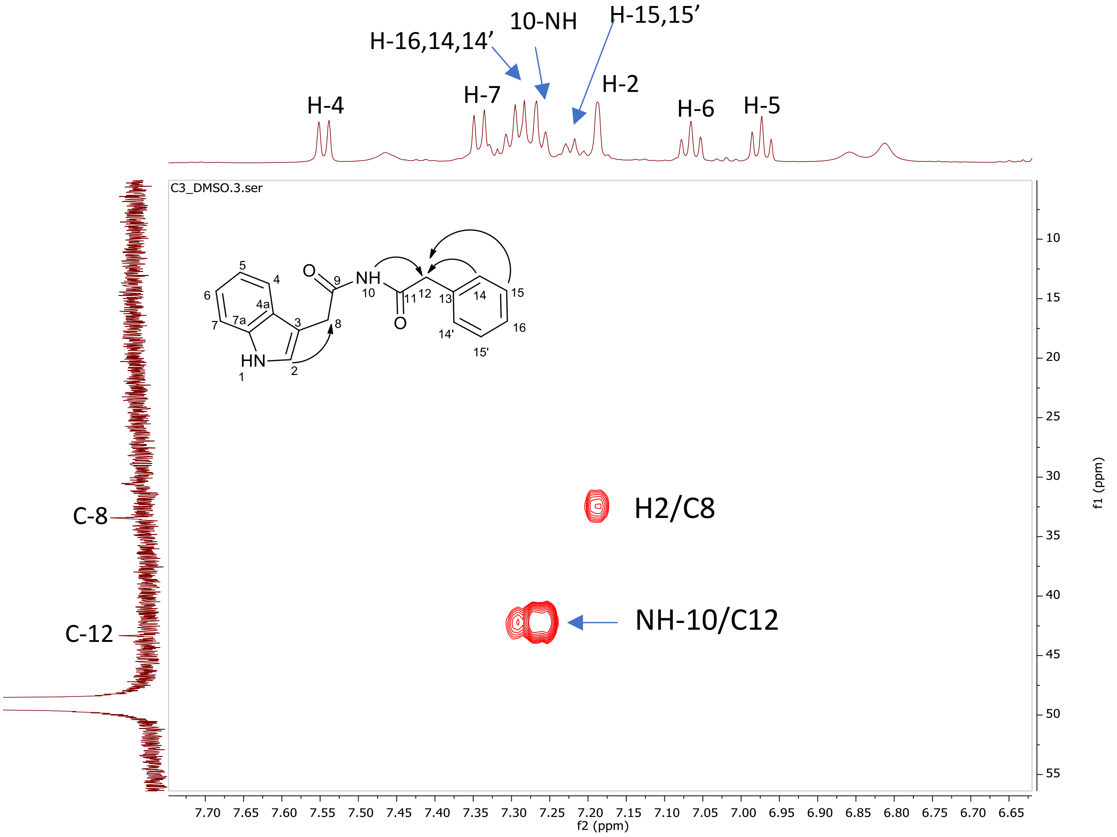

Figure S13. HMBC spectra of legonimide **1** showing correlations from NH-10 to C-12 while no correlation from NH-10 to C-8 signifying that the imide motif in **1** is a *cis-trans* conformer (DMSO-d_6)

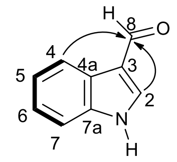

2

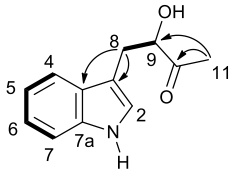

3

Table S4. Comparison of the ^{1}H and ^{13}C-NMR of **2-3** with the NMR data reported in literature[2,3] for 1H-indole-3-carbaldehyde and actinopolymorphol B, respectively [2–4] (CD_3OD, ^{1}H-NMR at 400 MHz, ^{13}C-NMR at 100 MHz)

No.	Compound **2**		1H-indole-3-carbaldehyde		Compound **3**		Actinopolymorphol B	
	^{13}C	^{1}H, mult. (*J*, Hz)	^{13}C	^{1}H, mult. (*J*, Hz)	^{13}C	^{1}H, mult. (*J*, Hz)	^{13}C	^{1}H, mult. (*J*, Hz)
2	139.9, CH	8.10, s	140.1, CH	8.04, s	124.6, CH	7.11, s	123.8, CH	7.14, s
3	123.7, C	-	119.7, C	-	112.3, C	-	117.4,C	-
4	120.0, CH	8.15, dd (dd,7.0,2.2)	123.8, CH	8.16, (dd,6.9,2.1)	119.3, CH	7.57(d,9.0)	118.6,CH	7.60(d,9.0)
5	123.7, CH	7.21, m	125.2, CH	7.22, m	119.7, CH	7.00(dt,0.8,.9.0)	118.9,CH	7.03(dt,0.8,.9.0)
6	122.3, CH	7.28, m	122.7, CH	7.27, m	122.4,CH	7.08(dt,0.8,.9.0)	121.5,CH	7.11(dt,0.8,.9.0)
7	113.2, CH	7.48, dd (dd,7.0,1.4)	113.4, CH	7.46, (dd,6.9,1.5)	111.0, CH	7.32(d,9.0)	111.4,CH	7.35(d,9.0)
8	187.6, C	-	187.8, C	-	30.6, CH_2	3.18, (dd,5.5,15.2), 3.05, (dd,7.2,15.2)	30, CH_2	3.21(dd,5.5,15.0), 3.09(dd,7.0,15.0)
4a	125.0, C	-	125.5, C	-	128.7,C	-	128.1,C	-
7a	138.8, C	-	138.7, C	-	138.0,C	-	137.2,C	-
8-OH	-	9.90, s	-	9.89, s	-	-	-	-
9	-	-	-	-	78.6, CH	4.42(dd,5.5,7.2)	77.9, CH	4.39(dd,5.5,7.0)
10	-	-	-	-	211.0,C	-	212.7,C	-
11	-	-	-	-	26.4,CH_3	2.10(s)	25.5,CH_3	2.14(s)

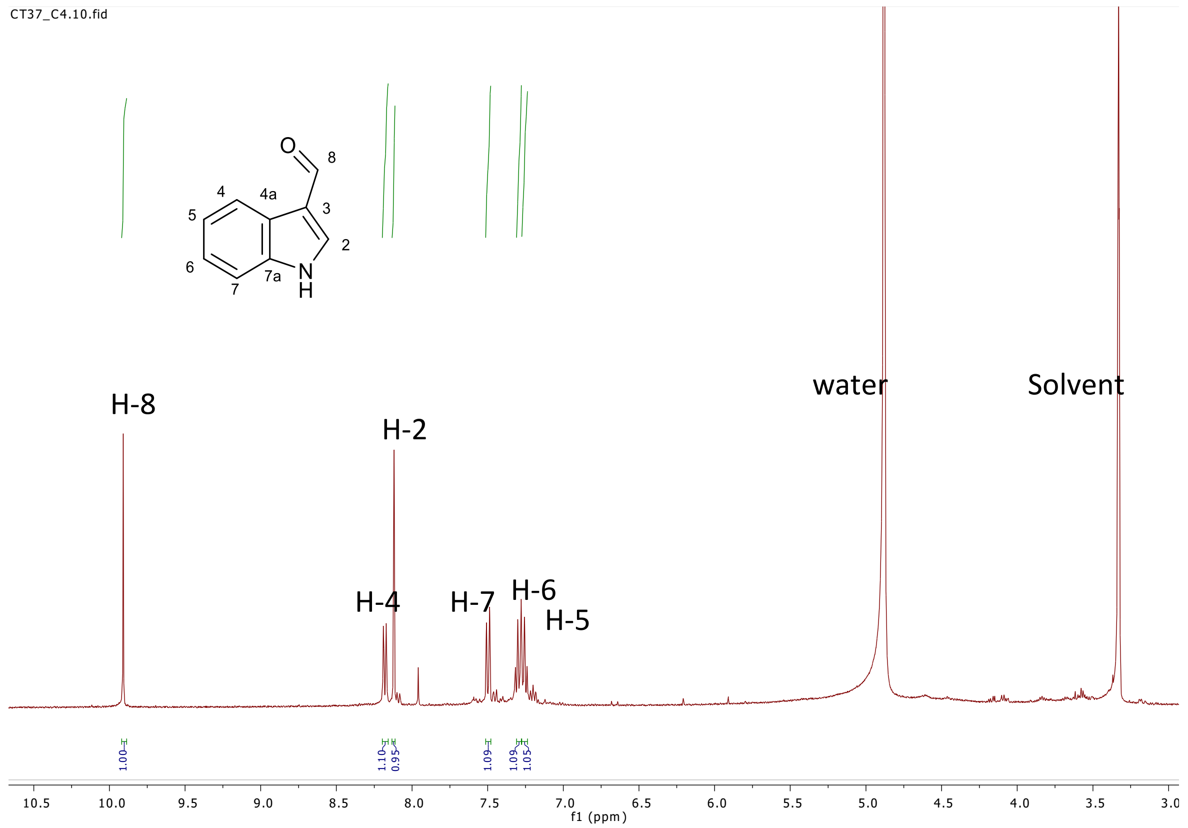

Figure S14. ^{1}H-NMR of **2** in CD_3OD at 600MHz

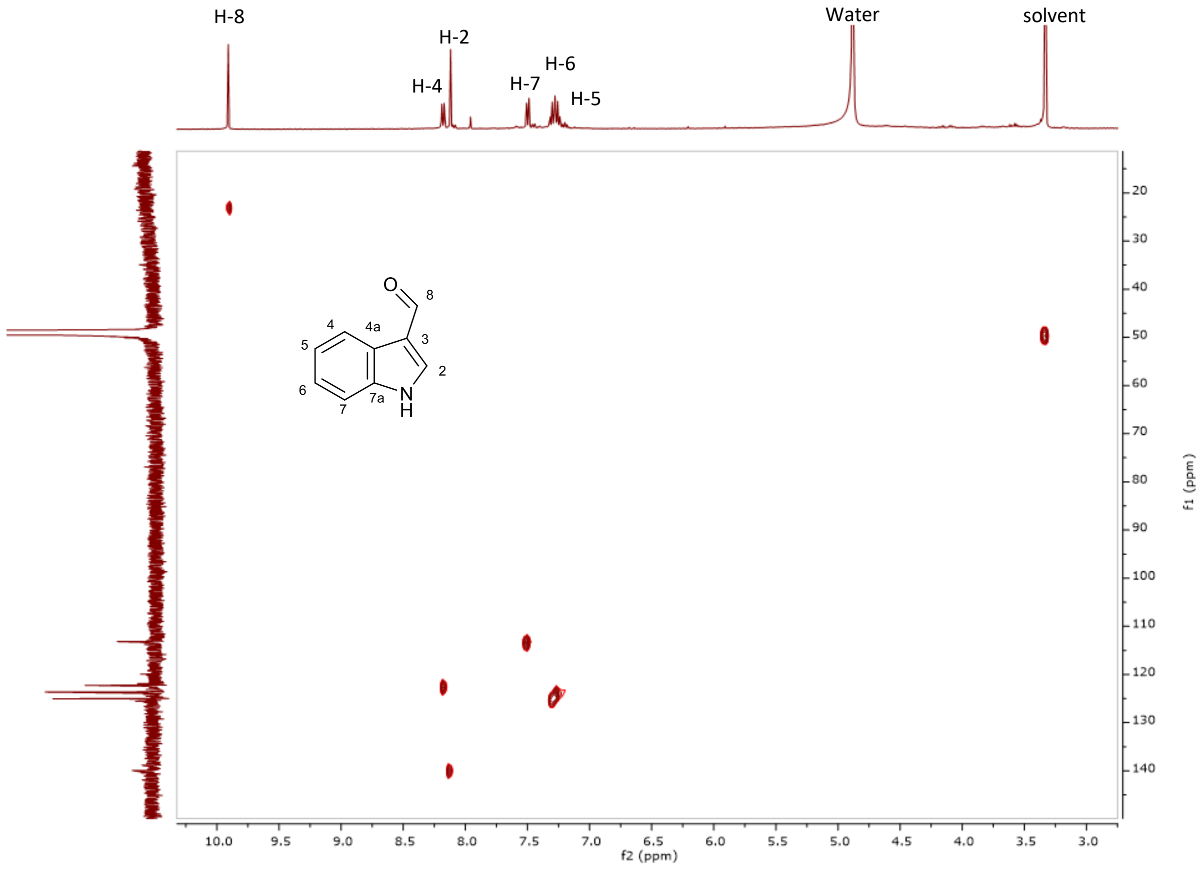

Figure S15. HSQC of **2** in CD_3OD at 600MHz

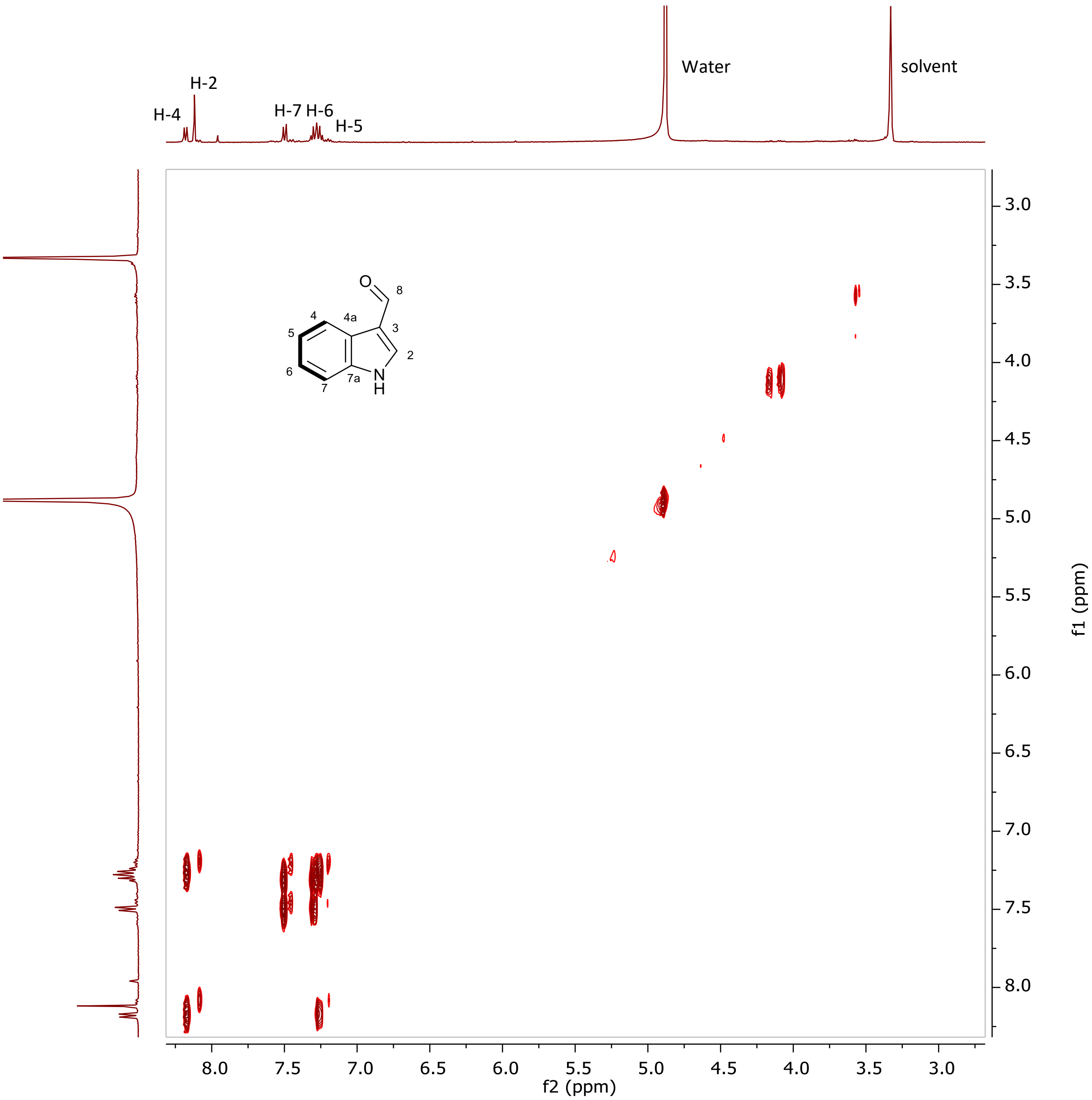

Figure S16. COSY of **2** in CD_3OD at 600MHz

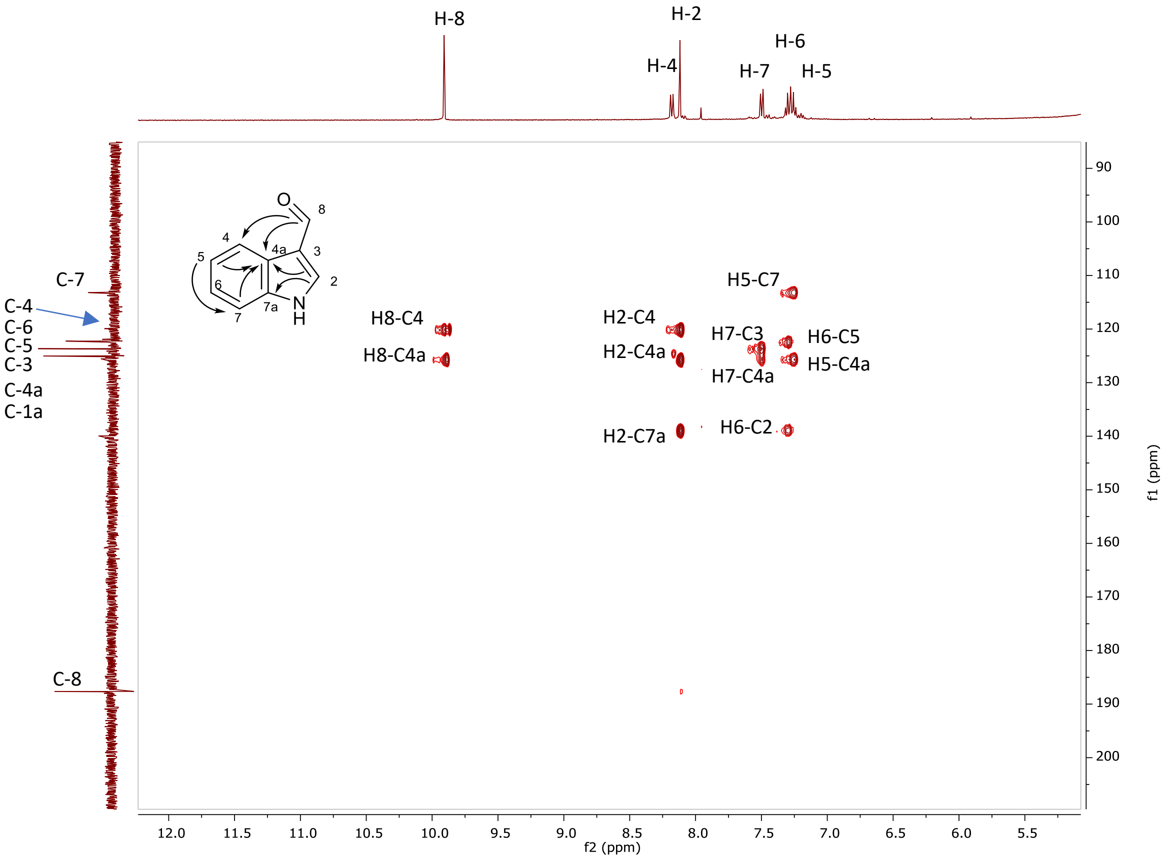

Figure S17. HMBC of **2** in CD_3OD at 600MHz

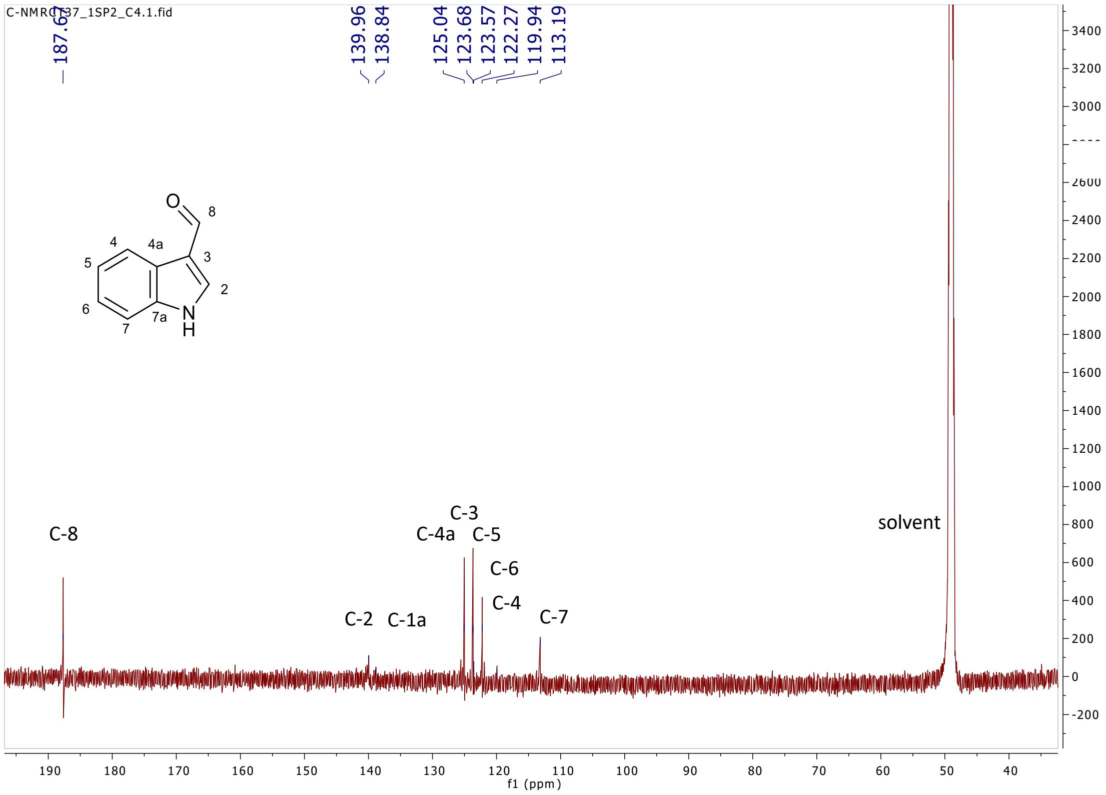

Figure S18. ^{13}C-NMR of **2** in CD_3OD at 600MHz

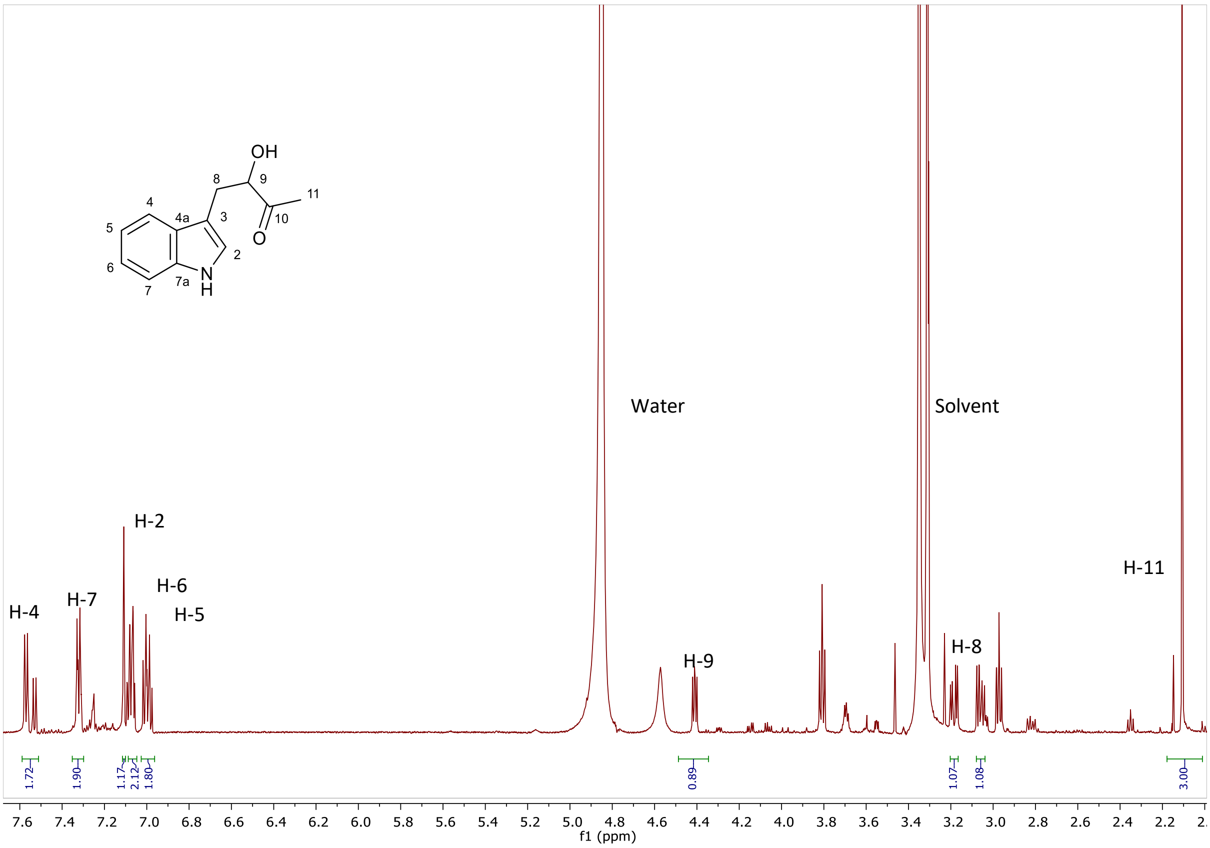

Figure S19. ^{1}H-NMR of **3** in CD_3OD at 600MHz

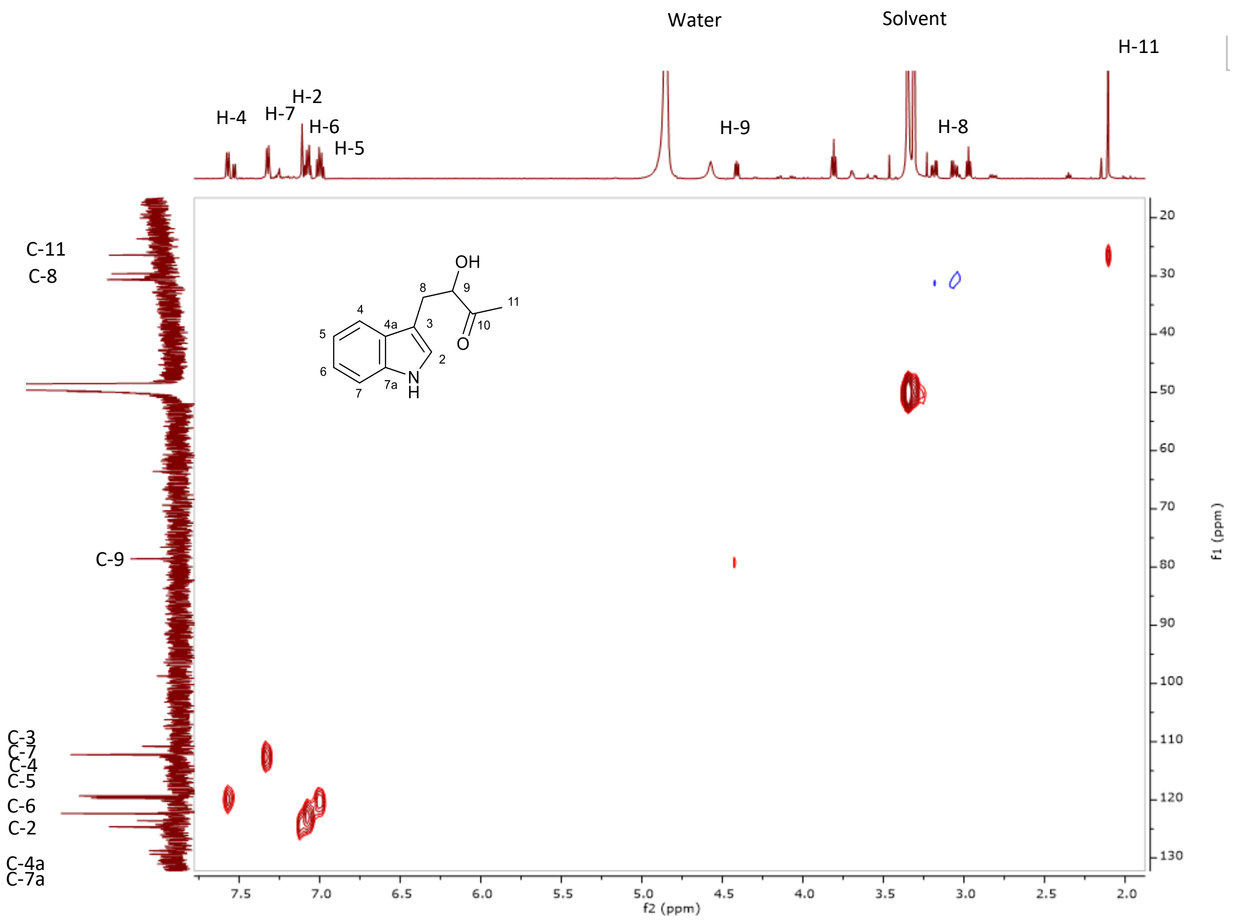

Figure S20. HSQC of **3** in CD_3OD at 600MHz

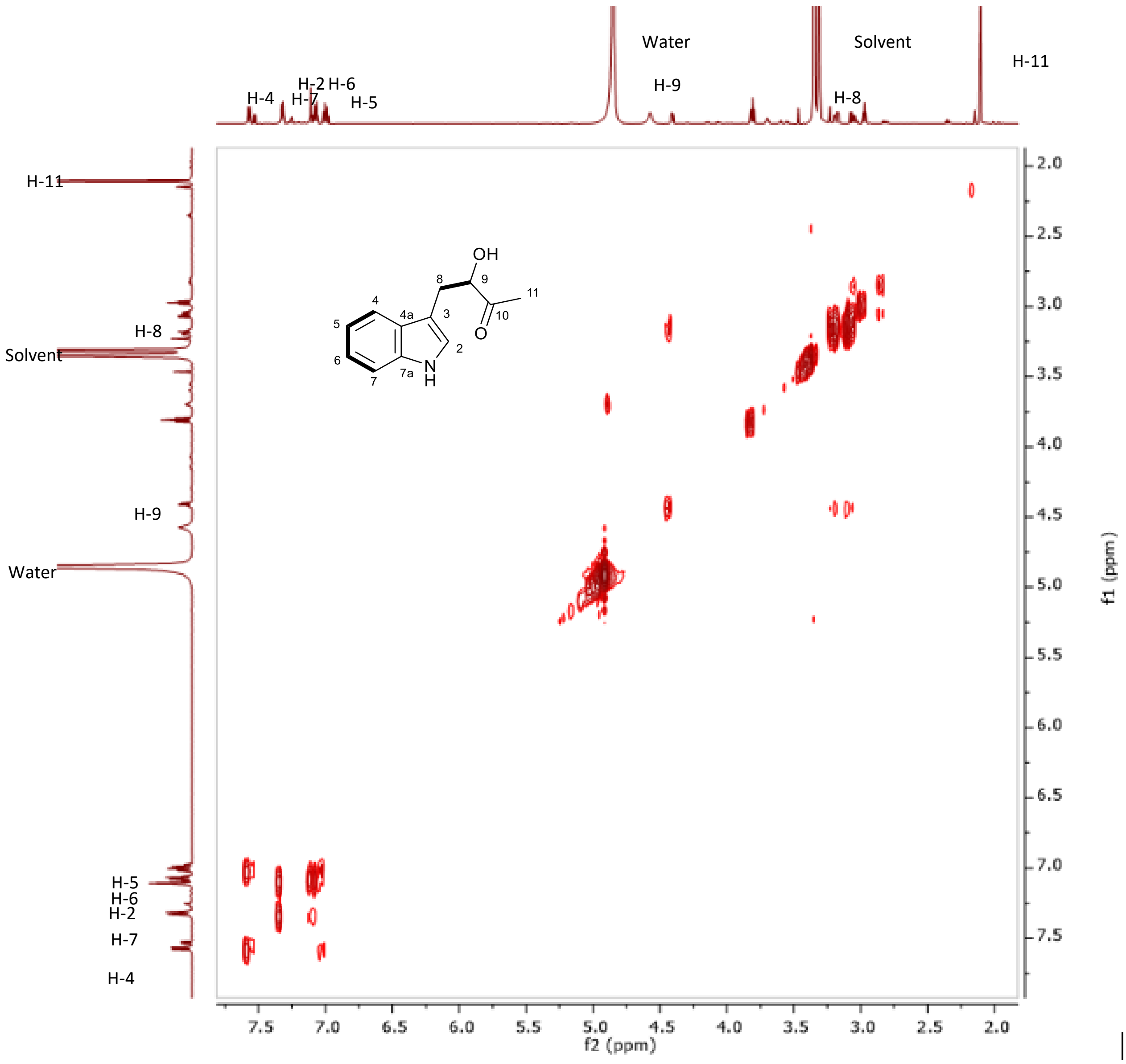

Figure S21. COSY of **3** in CD_3OD at 600MHz

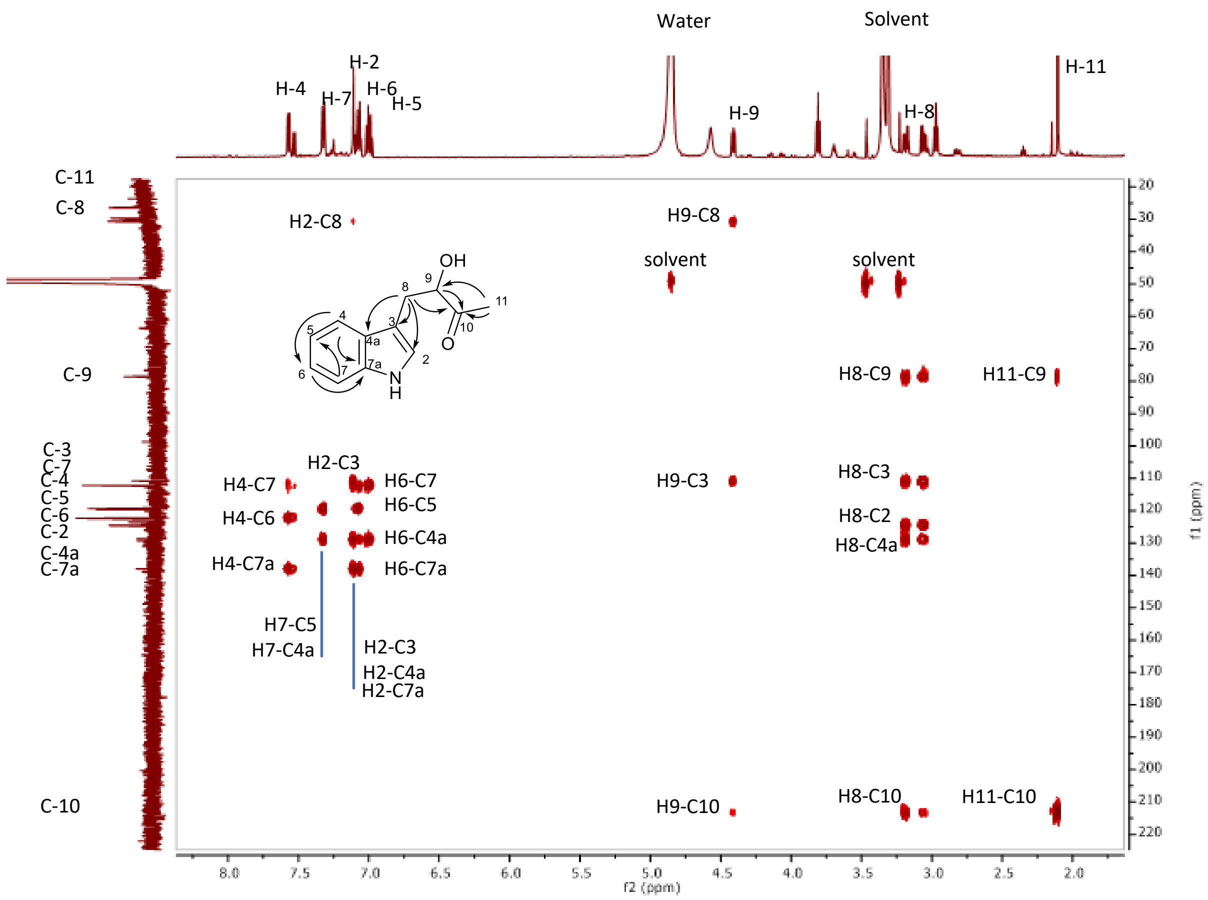

Figure S22. HMBC of **3** in CD_3OD at 600MHz

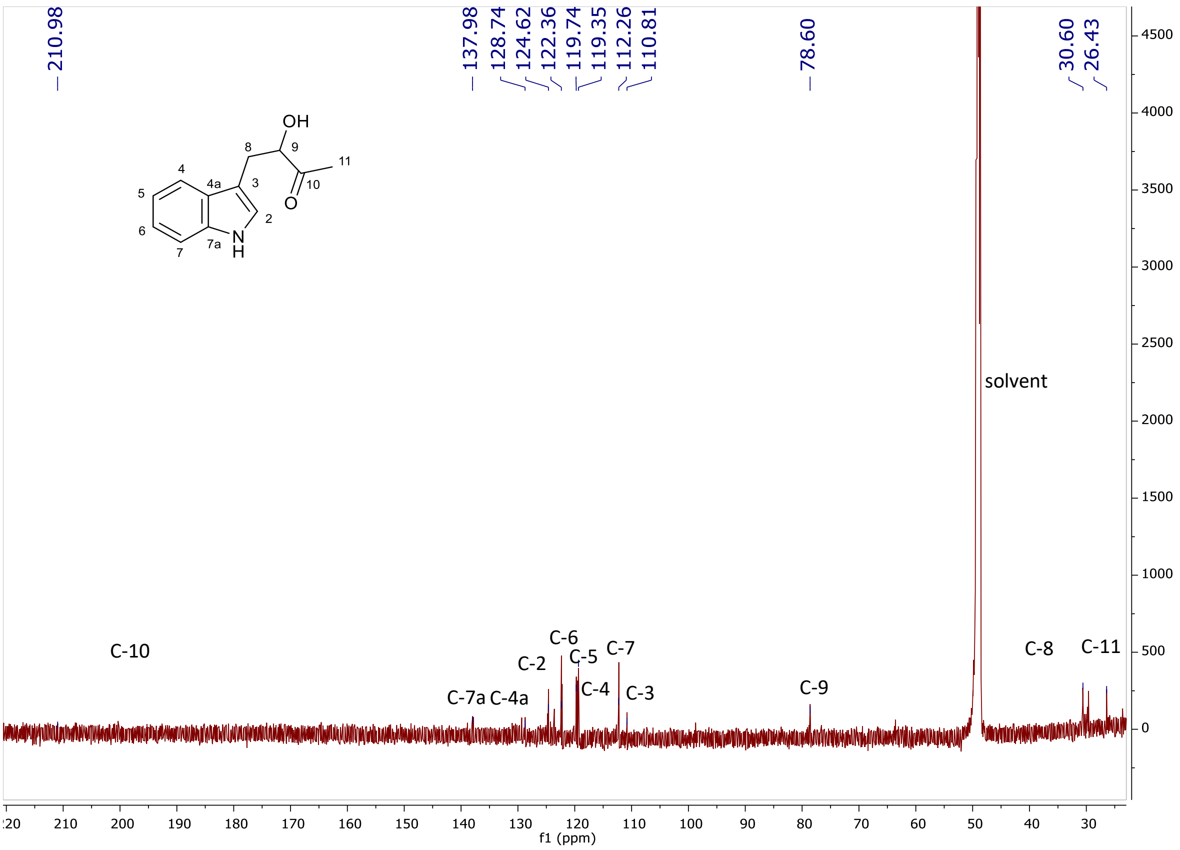

Figure S23. ^{13}C-NMR of **3** in CD_3OD at 600MHz

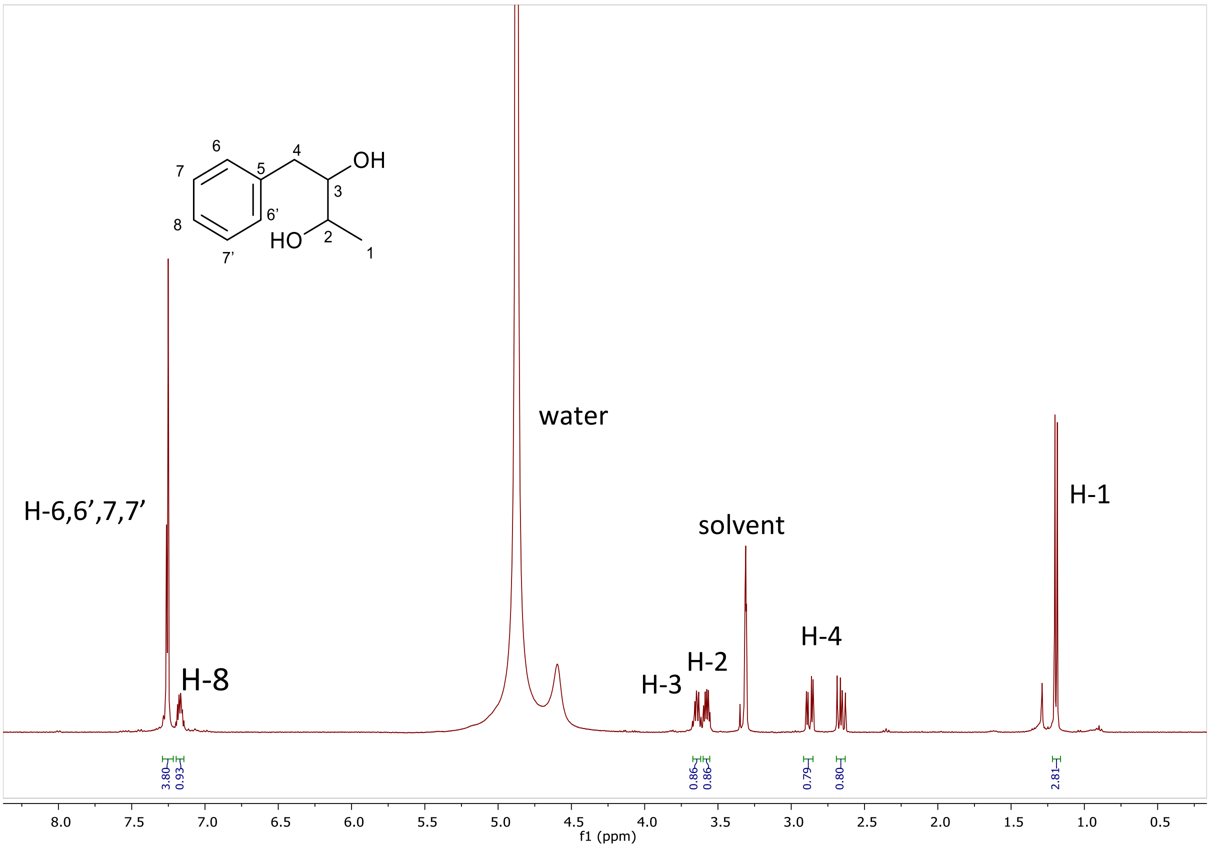

Figure S24. ^{1}H-NMR of **4** in CD_3OD at 600MHz

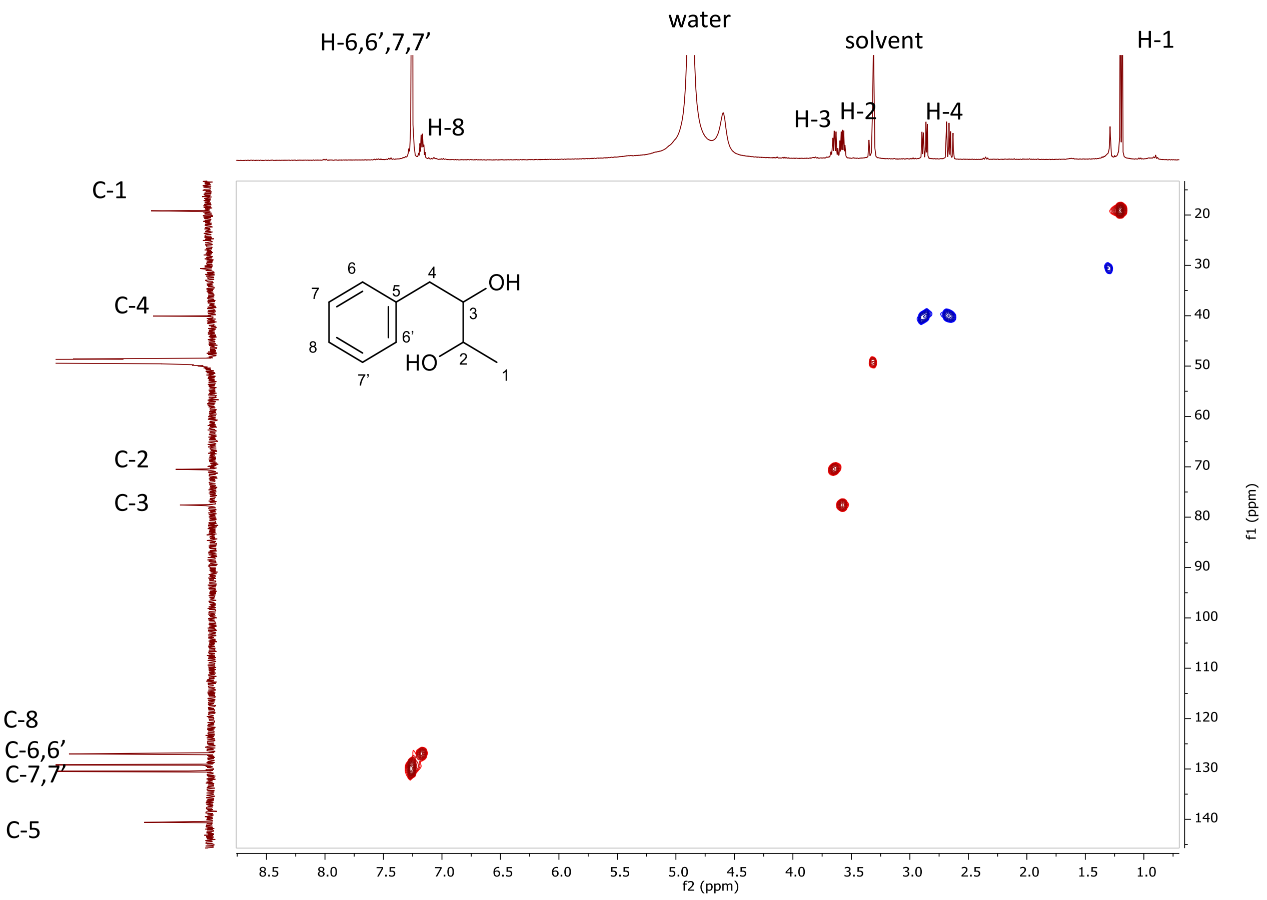

Figure S25. HSQC of **4** in CD_3OD at 600MHz

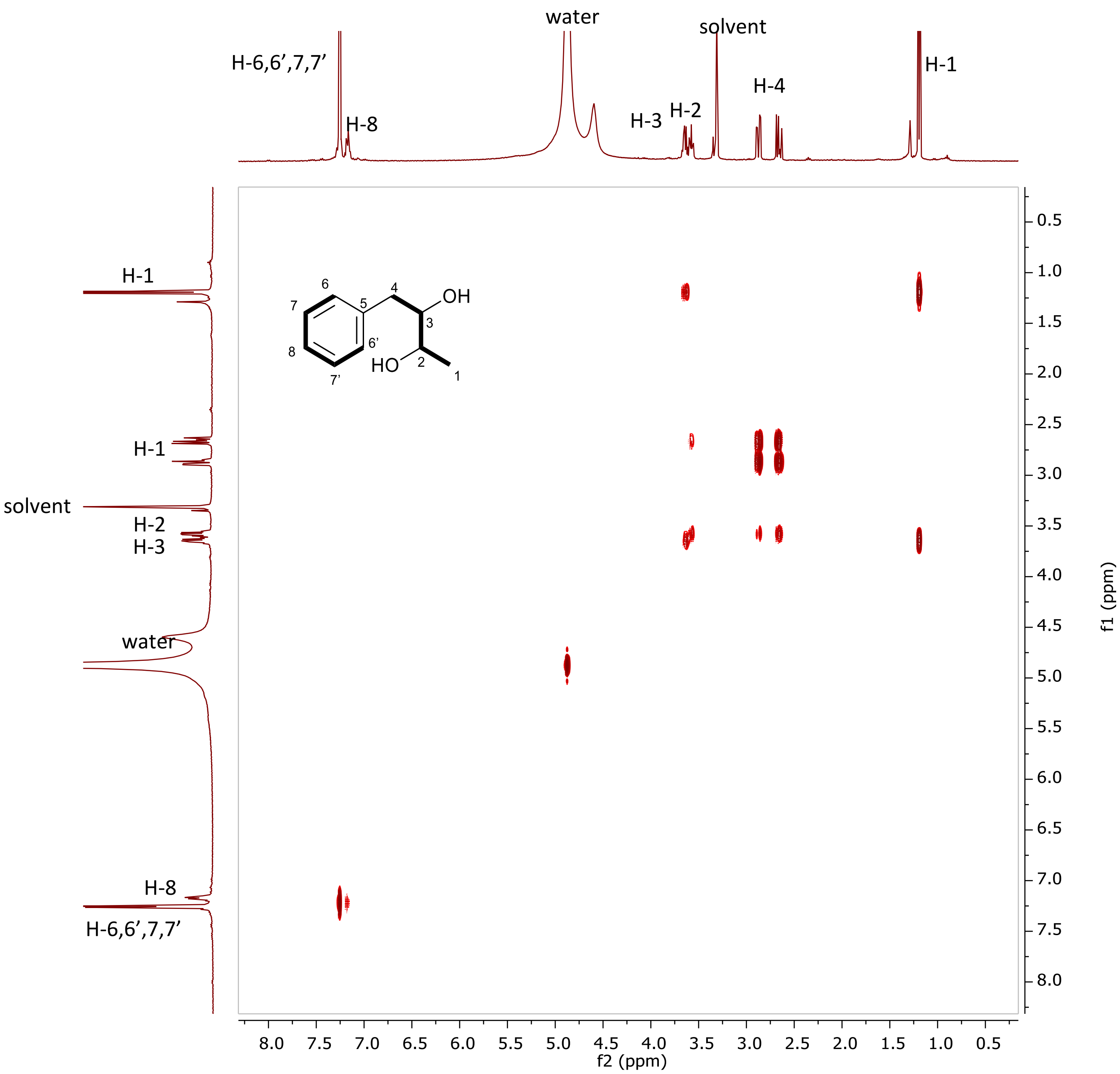

Figure S26. COSY of **4** in CD_3OD at 600MHz

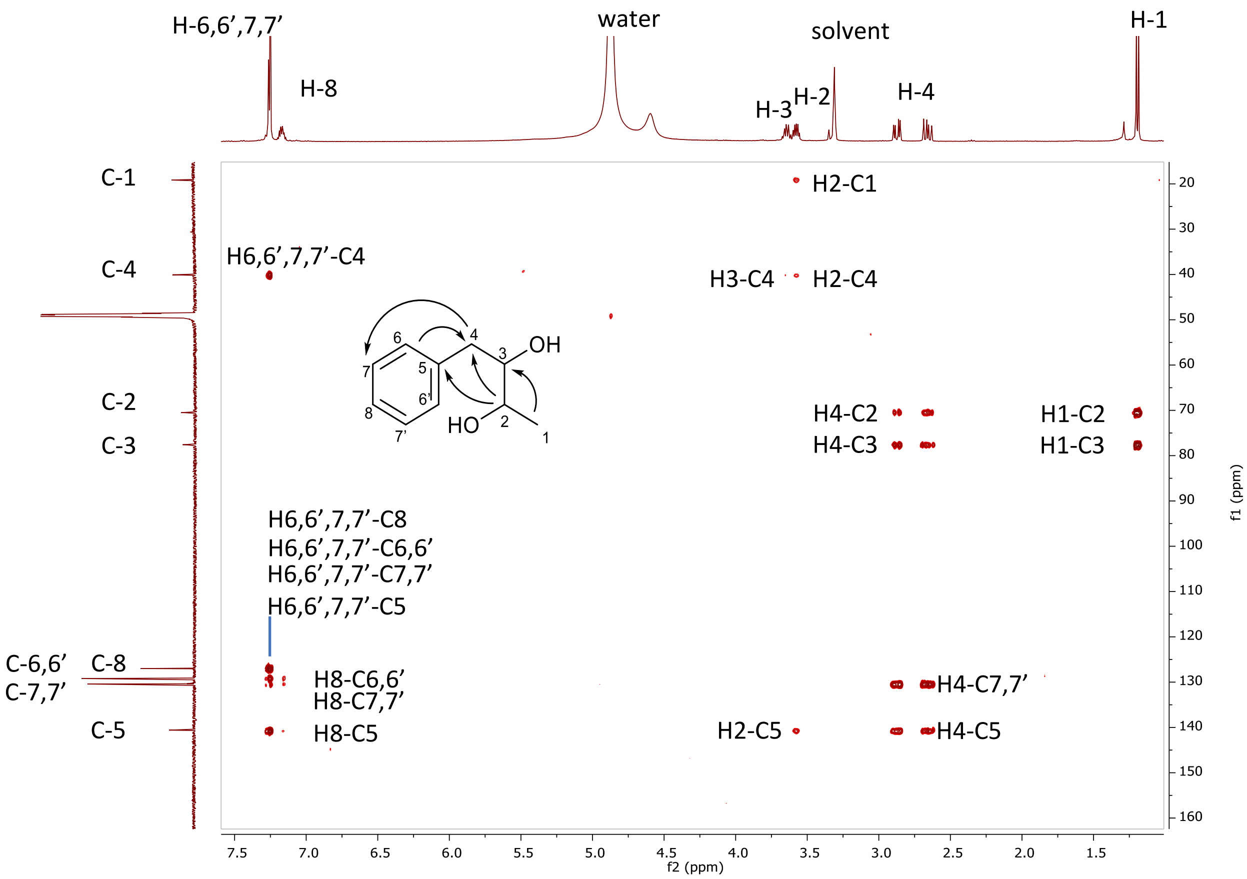

Figure S27. HMBC of **4** in CD_3OD at 600MHz

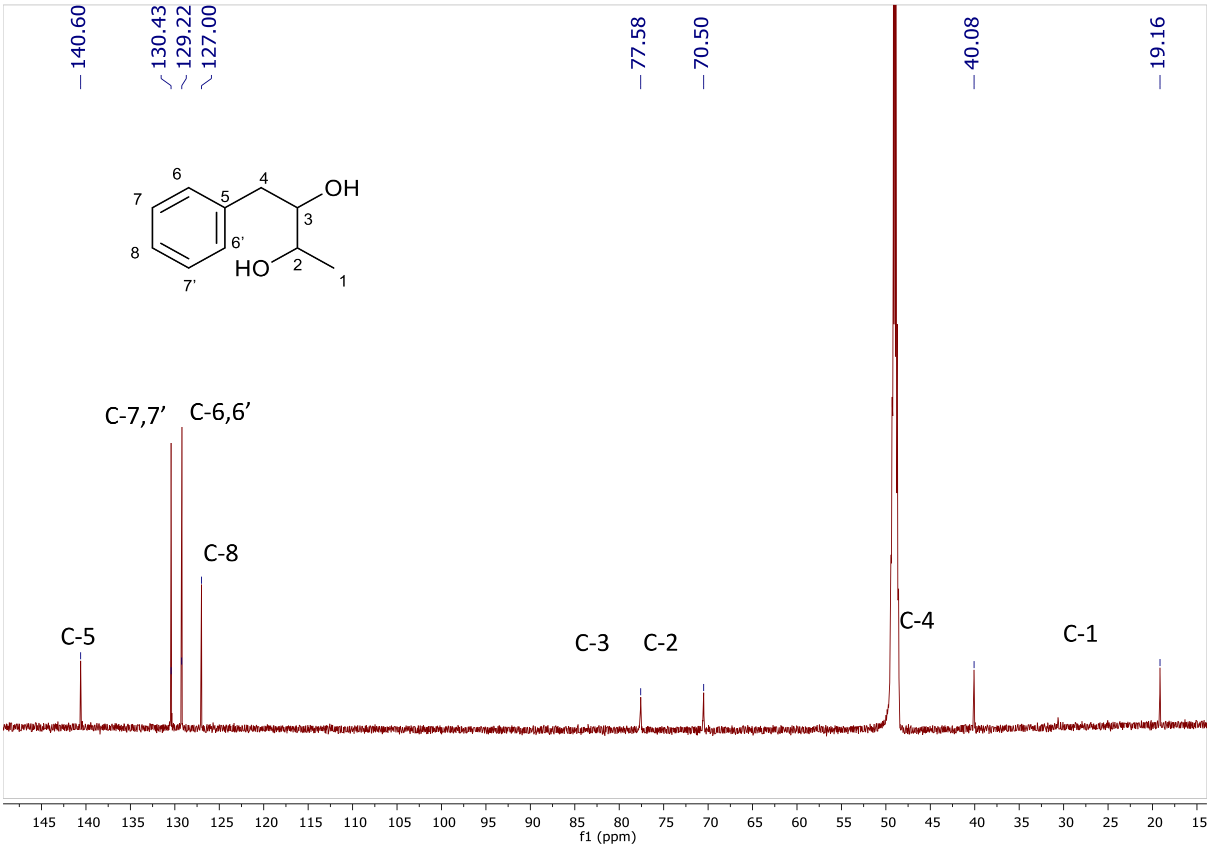

Figure S28. ^{13}C-NMR of **4** in CD_3OD at 600MHz

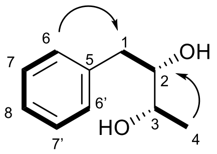

4

threo isomers

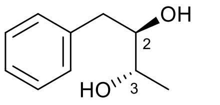

(2S,3R)-1-phenylbutane-2,3-diol

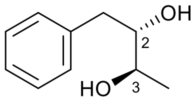

(2R,3S)-1-phenylbutane-2,3-diol

erythro isomers

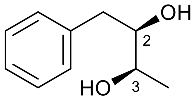

(2S,3S)-1-phenylbutane-2,3-diol

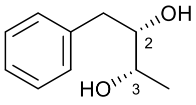

(2R,3R)-1-phenylbutane-2,3-diol

Table S5. ^{1}H (400MHz) and ^{13}C-NMR (100MHz) of compound **4** ($CDCl_3$) in comparison with the NMR data of the four stereoisomers reported in literature [5,6] (300MHz, $CDCl_3$)

No.	Compound **4**		*Threo* isomers (2S,3R)-1-phenylbutane-2,3-diol (2R,3S)-1-phenylbutane-2,3-diol		*Erythro* isomers (2S,3S)-1-phenylbutane-2,3-diol (2R,3R)-1-phenylbutane-2,3-diol	
	^{13}C	^{1}H, mult. (*J*, Hz)	^{13}C	^{1}H, mult. (*J*, Hz)	^{13}C	^{1}H, mult. (*J*, Hz)
4	19.2	1.19 (d, 3H)	17.13	1.16 (d,3H, *J*=7)	19.35	1.26 (d,3H, *J*=7)
1	40.1	2.67(dd,1H), 2.87(dd,1H)	38.2	2.5-2.9 (4H, m)	39.72	2.6-3.0 (2H, m)
2	77.6	3.57 (m, 1H)	75.91	3.72 (m,2H)	75.56	3.5-3.7 (m,2H)
3	70.5	3.65 (q, 1H)	69.95		69.73	
6,6',7,7'	129.2, 130.4	7.16-7. 31 (m, 5H)	126.56, 129.38	7.15-7.35 (m,5H)	126.18, 129.32	7.20-7.4 (m,5H)
8	127.0	-	128.67	-	128.29	-
5	140.6	-	138.48	-	138.43	-
OH		2.02 (br,1H， s)				2.16 (br,1H， s)
		2.23 (br,1H， s)				2.40 (br,1H， s)

Table S6. Optical rotation of compound **4** in comparison with the optical rotation reported for the four stereoisomers in literature [5,6]

	Sample name	Optical Rotation
Threo isomers	(2R,3S)-1-phenylbutane-2,3-diol	$[\alpha]^{25}_{D}$ +41.99 (*c* 1.124 , $CHCl_3$)
	(2S,3R)-1-phenylbutane-2,3-diol	$[\alpha]^{25}_{D}$ -41.94 (*c* 1.130 , $CHCl_3$)
Erythro isomers	(2R,3R)-1-phenylbutane-2,3-diol	$[\alpha]^{24}_{D}$ +35.53 (*c* 1.162 , $CHCl_3$)
	(2S,3S)-1-phenylbutane-2,3-diol	$[\alpha]^{24}_{D}$ -32.58 (*c* 1.074 , $CHCl_3$)
	Compound **4**	$[\alpha]^{25}_{D}$ +14.1 (*c* 0.500 , $CHCl_3$)

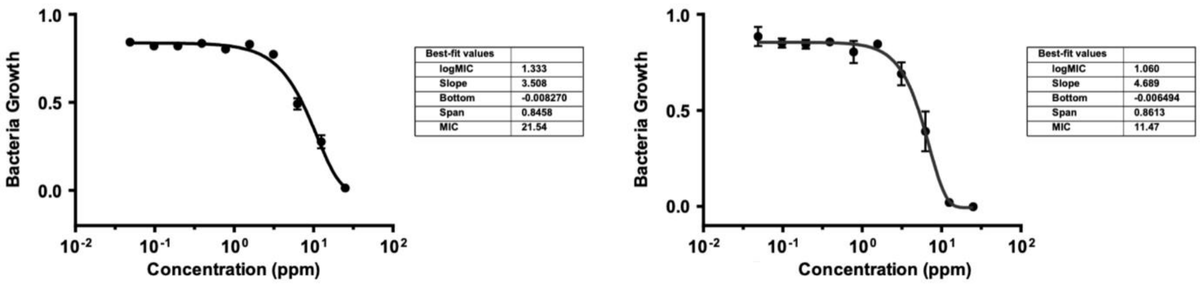

Figure S29. MIC curve of **1** and **2** against *Candida albicans* ATCC 1023

References:

1. Parra, R.D.; Furukawa, M.; Gong, B.; Zeng, X.C. Energetics and cooperativity in three-center hydrogen bonding interactions. I. Diacetamide-X dimers (X=HCN, CH3OH). *J. Chem. Phys.* **2001**, *115*, 6030–6035.
2. Yu, L.; Liu, J.; Yu, L.; Chen, L.; Qiu, F. Chemical Constituents of Seed Oil Leavings of Xanthoceras sorbifolia. *Chem. Nat. Compd.* **2018**, *54*, 769–771.
3. Huang, S.X.; Powell, E.; Rajski, S.R.; Zhao, L.X.; Jiang, C.L.; Duan, Y.; Xu, W.; Shen, B. Discovery and total synthesis of a new estrogen receptor heterodimerizing actinopolymorphol A from actinopolymorpha rutilus. *Org. Lett.* **2010**, *12*, 3525–3527.
4. Netz, N.; Opatz, T. Marine indole alkaloids. *Mar. Drugs* **2015**, *13*, 4814–4914.
5. Awano, K.I.; Yanai, T.; Watanabe, I.; Takagi, Y.; Kitahara, T.; Mori, K. Synthesis of all Four Possible Stereoisomers of 1-Phenyl-2,3-Butanediol and Both Enantiomers of 3-Hydroxy-4-Phenyl-2-Butanone to Determine the Absolute Configuration of the Natural Constituents. *Biosci. Biotechnol. Biochem.* **1995**, *59*, 1251–1254.
6. Cartus, A.T.; Stegmüller, S.; Simson, N.; Wahl, A.; Neef, S.; Kelm, H.; Schrenk, D. Hepatic Metabolism of Carcinogenic β-Asarone. *Chem. Res. Toxicol.* **2015**, *28*, 1760–1773.
